# MiR-140-3p regulates axonal motor protein KIF5A and contributes to axonal transport degeneration in SMA

**DOI:** 10.1038/s41420-025-02663-x

**Published:** 2025-10-07

**Authors:** Markella Baklou, Valeria Valsecchi, Giusy Laudati, Xhesika Kolici, Paola Brancaccio, Nunzia De Iesu, Serenella Anzilotti, Federica Cieri, Giuseppe Pignataro

**Affiliations:** 1 Division of Pharmacology, Department of Neuroscience, Reproductive and Dentistry Sciences, School of Medicine, Federico II University of Naples, Naples, Italy; 2 School of Advanced Studies, Center for Neuroscience, University of Camerino, Camerino, Italy; 3 IRCCS Synlab SDN, Naples, Italy; 4 Department of Human Sciences and Quality of Life Promotion, Università San Raffaele, Rome, Italy; 5 Present Address: Institute of Medical Sciences, School of Medicine, Medical Sciences and Nutrition, University of Aberdeen, Aberdeen, Scotland

**Keywords:** Molecular biology, Neuroscience

## Abstract

Spinal muscular atrophy (SMA) is a paediatric neuromuscular disease caused by alterations of the survival motor neuron (*SMN*) gene, which results in progressive degeneration of motor neurons (MNs). Although effective treatments for SMA patients has been recently developed, the molecular pathway involved in selective MNs degeneration has not been yet elucidated. Disruption of axonal transport is a common feature of motor neuron diseases (MNDs); specifically, mutations at the C-terminal of the kinesin KIF5A, have been linked to neurodegenerative disorders involving MNs degeneration such as amyotrophic lateral sclerosis (ALS). Therefore, the present study attempts to investigate potential alterations of the axonal transport complex that includes KIF5A in a SMA mouse model. We demonstrated that KIF5A is downregulated in the spinal cord of SMA mice both in early and late phases of the disease. A miRNA-based strategy was developed in the attempt to prevent KIF5A downregulation, thus restoring its physiological levels. Indeed, we demonstrated that miR-140-3p was up-regulated in the spinal cord of SMA mice during disease progression and was able to negatively modulate KIF5A expression. Furthermore, the intracerebroventricular injection of an antagomir molecule, able to block miR140-3p function, resulted in a reduction of SMA severity in terms of improved behavioural performance. Based on these results, we indicated KIF5A as a distinctive mechanism of MNDs progression and suggested that developing a strategy able to prevent KIF5A downregulation could be beneficial, not only in SMA but also in other neurodegenerative diseases.

## Introduction

Spinal Muscular Atrophy (SMA) is the most common genetic cause of infant mortality [1]. Most forms of SMA are caused by homozygous mutations in *Survival Motor Neuron 1* (*SMN1)* gene [2]. A second gene, *SMN2*, is a paralogue gene present in a variable number of copies and, because of a nucleotide change in a splicing site, it can only partially compensate for *SMN1* loss [3]. Accordingly, the number of *SMN2* copies directly correlates with disease severity [3]. On the basis of *SMN2* number of copies and the age of onset, we classify four clinical SMA groups (type 1-4), which inversely correlate with the number of *SMN2* copies and the SMN protein levels [4]. SMN protein is ubiquitously expressed throughout the body [5]; with important role in transcriptional processes, in protein translation, autophagy and ubiquitination [6]. Recently, FDA and EMA have approved three SMN-dependent treatments for SMA [7]: the antisense oligonucleotides Nusinersen, the pyridazine derivative Risdiplam, which modify *SMN2* splicing, and *SMN1* replacement by gene therapy Onasemnogene abeparvovec. Nevertheless, all treatments have limitations, such as a narrow therapeutic window, a sparse efficacy and targeting limited to specific tissues [8]. Moreover, they are usually administered intrathecally and not accessible to all patients in terms of costs [9]. Although several studies have been carried out in the last decade, the molecular pathway involved in selective MNs degeneration has not been yet elucidated.

Notably, disruption of axonal transport is a common feature of motor neuron diseases (MNDs), with the kinesin proteins that play a central role for the transport machinery. In particular, the mammalian kinesin superfamily (KIFs) is comprised of 45 genes, most of which are expressed in the brain. They can be classified into 15 families and each kinesin has a characteristic velocity that ranges from 0.2 μm/sec to 1.5 μm/sec that corresponds to fast axonal transport [10]. In particular, kinesin 1 superfamily composed by KIF5A, KIF5B and KIF5C isoforms, is the most abundant and mediate the anterograde transport towards the plus end of the microtubules to the axon terminal [11]. Unlike some other kinesins, KIF5 can form dimers and in some cases, tetramers with two kinesin light chains (KLCs). KIF5A and KIF5C are exclusively expressed in the brain and spinal cord (SC), with a pan-neuronal expression [12], whereas KIF5B is ubiquitously expressed, including neurons and glial cells [13].

The function of KIF5A, the longest of the three kinesin 1 superfamily, has been linked with neurodegenerative diseases. In particular, different types of KIF5A mutations (missense, non-sense, splicing, frameshift) have been reported, and their location seems to dictate the phenotype. Motor and stalk domain mutations have been previously associated with hereditary spastic paraplegia (HSP) type 10 (SPG10) [14] and Charcot-Marie-Tooth (CMT) type 2 [15], two diseases characterised by anterograde neurodegeneration, as well as with atypical motor syndromes [16]. Furthermore, mutations in the C-terminal are causative of neonatal intractable myoclonus (NEIMY), a severe congenital syndrome characterised by myoclonic seizures with evidence of mitochondrial dysfunction [17, 18] and amyotrophic lateral sclerosis (ALS), the most common adult-onset MND.

The KIF5A C-terminal domain is responsible for the transport of important cargoes, such as neurofilaments, whose accumulation is a hallmark in ALS [19], RNA granules [13, 20] lysosomes [21], amyloid precursor protein (APP) vesicles, and mitochondria [22], mainly via the adaptor protein complex MIRO1/TRAK1/2 [23–29].

In the light of these premises, the present study attempted to investigate potential alteration of KIF5A in a SMA mouse model and to explore a miRNA-based strategy to reduce SMA progression.

## Results

### KIF5A is downregulated in the spinal cord of SMA mice

**Fig. 1:**
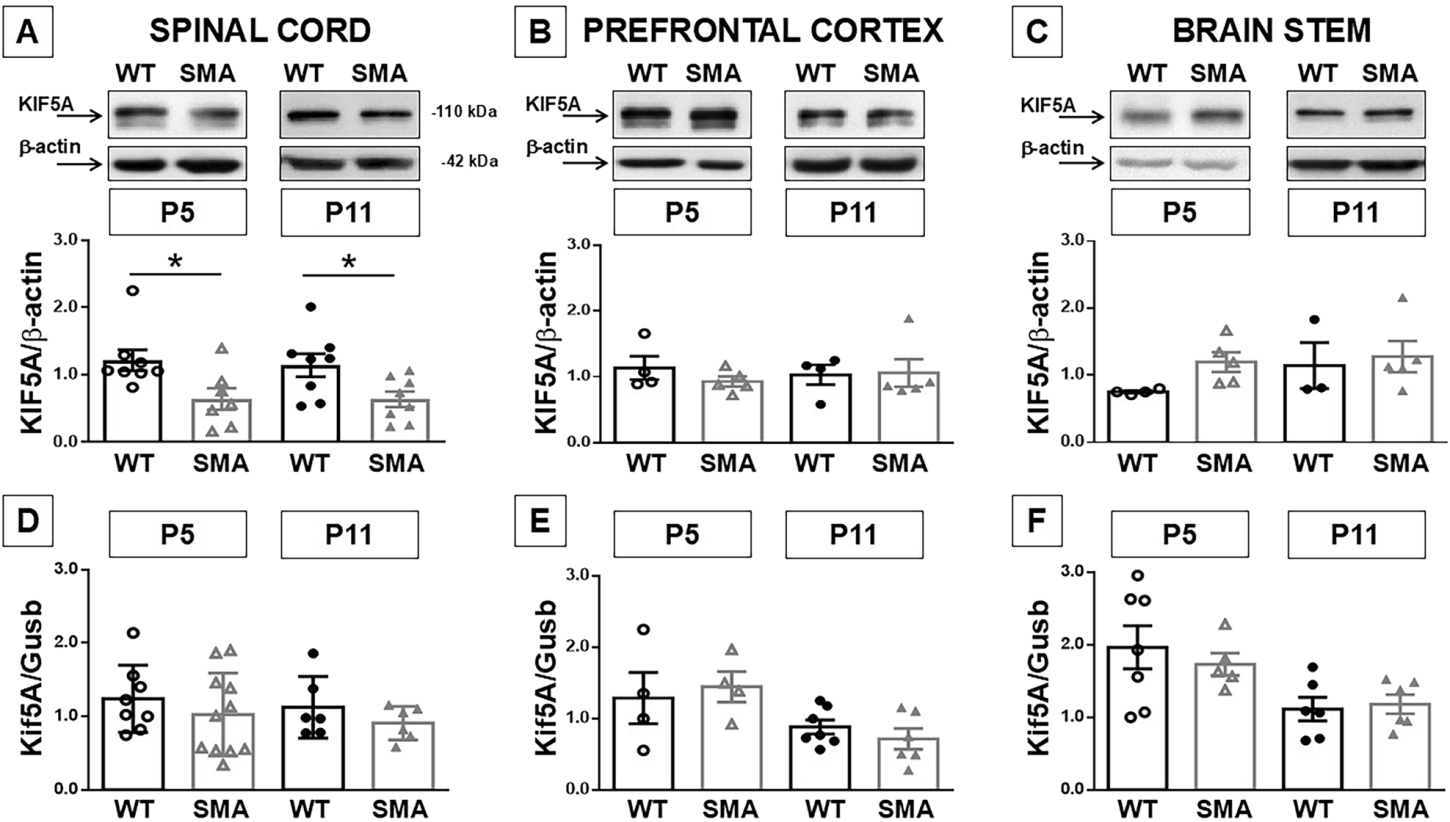
KIF-5A expression in spinal cord, prefrontal cortex and brain stem of early and late symptomatic SMA mice. Western blot (**A**–**C**) and mRNA expression (**D**–**F**) of KIF5A in spinal cord (**A**, **D**), prefrontal cortex (**B**, **E**) and brain stem (**C**, **F**) of WT (black dots) and SMA mice (gray triangles), 5 (P5, empty dots and triangles) and 11 (P11, full dots and triangles) days after birth. Each column represents the mean ± SEM. Each point indicates a sample. *p < 0.05 vs age-matched WT by one-way ANOVA analysis followed by Bonferroni test.

To investigate if KIF5A might play a role in SMA pathology, KIF5A expression levels were measured in prefrontal cortex, brain stem and spinal cord of early (P5) and late (P11) symptomatic *Smn*^*−/−*^*; hSMN2*^*+/+*^*; hSMN*Δ*7*^*+/+*^ (SMA) mice and compared these values to those of age-matched healthy controls (*Smn*^*+/+*^*; hSMN2*^*+/+*^*; hSMN*Δ*7*^*+/+*^; wild-type [WT]). Interestingly, WB analysis showed a strong down-regulation of 50% of KIF5A protein only in the spinal cord of SMA mice compared to age-matched control pups at both ages considered (Fig. 1A). Prefrontal cortex and brain stem did not show significant differences in KIF5A expression among genotypes during disease progression (Fig. 1B, C). By contrast, KIF5A downregulation observed in the spinal cord was not mediated at transcriptional levels as resulted from qPCR analysis, that did not show variations in all the CNS regions considered (Fig. 1D–F).

### KIF5A interactor proteins did not change in the spinal cord of SMA mice

**Fig. 2:**
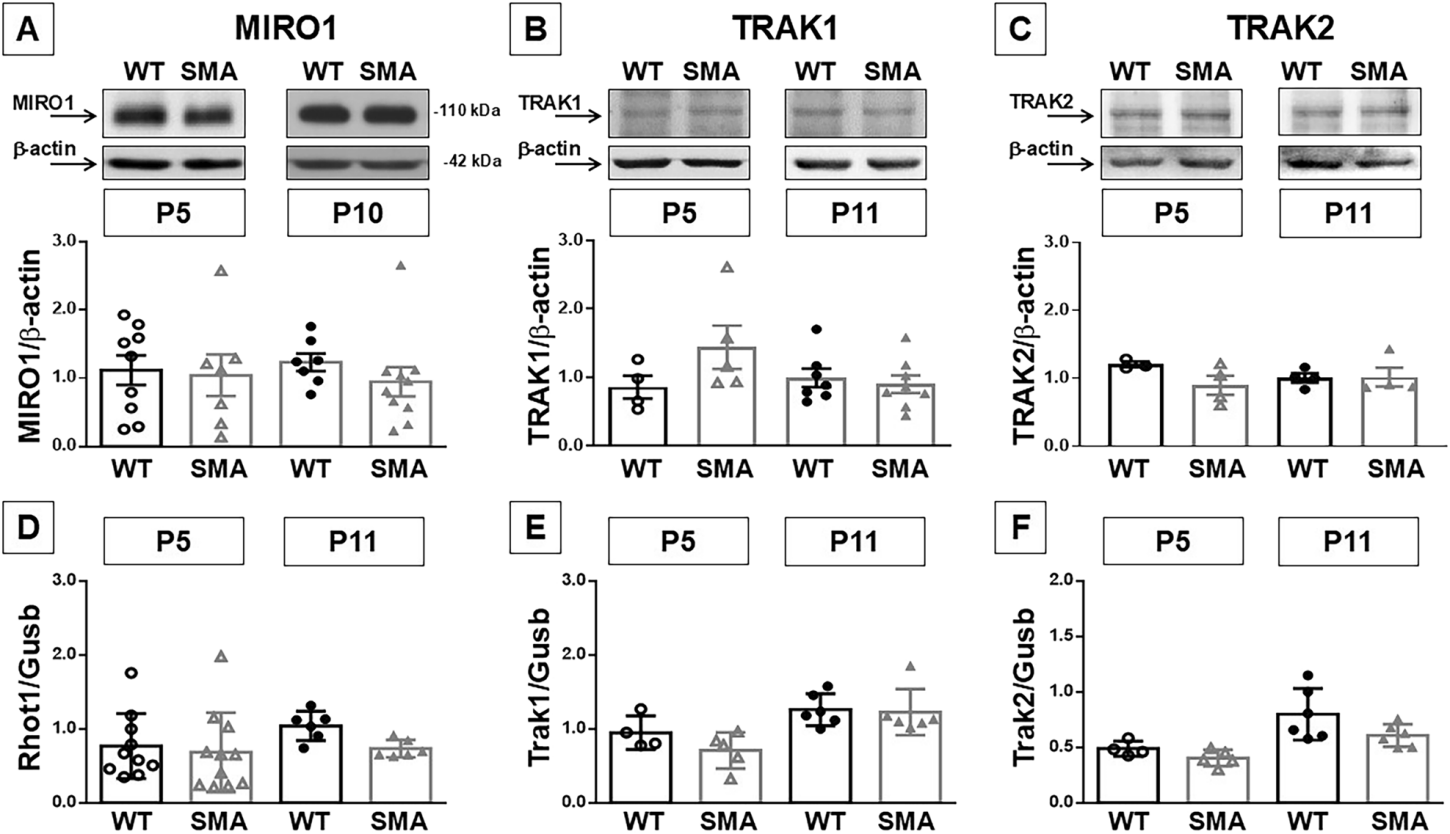
MIRO1, TRAK1 and TRAK2 expression in the spinal cord of early and late symptomatic SMA mice. Western blot (**A**–**C**) and mRNA expression (**D**–**F**) of MIRO1 and *Rhot1*gene (**A**, **D**), TRAK1 and *Trak1* gene (**B**, **E**), and TRAK2 and *Trak2* gene (**C**, **F**) in the spinal cord of WT (black dots) and SMA mice (gray triangles), 5 (P5, empty dots and triangles) and 11 (P11, full dots and triangles) days after birth. Each column represents the mean ± SEM. Each point indicates a sample.

In order to verify whether KIF5A downregulation reduces also the proteins interactions in which it is involved for axonal transport, the expression levels of TRAK1 Rho GTPase and MIRO1 were evaluated by WB and qPCR analysis. Surprisingly, MIRO1 protein levels did not change in the spinal cord of SMA mice during disease progression (Fig. 2A). Moreover, no changes in the expression were observed for MIRO1-kinesin1 adaptor proteins TRAK1 and TRAK2 (Fig. 2B, C). Analogously, no transcriptional variations were observed for *Rhot1*, the gene coding for MIRO1, *Trak1* and *Trak2* in the spinal cord of SMA compared to WT pups at the ages considered (Fig. 2D–F).

**Fig. 3:**
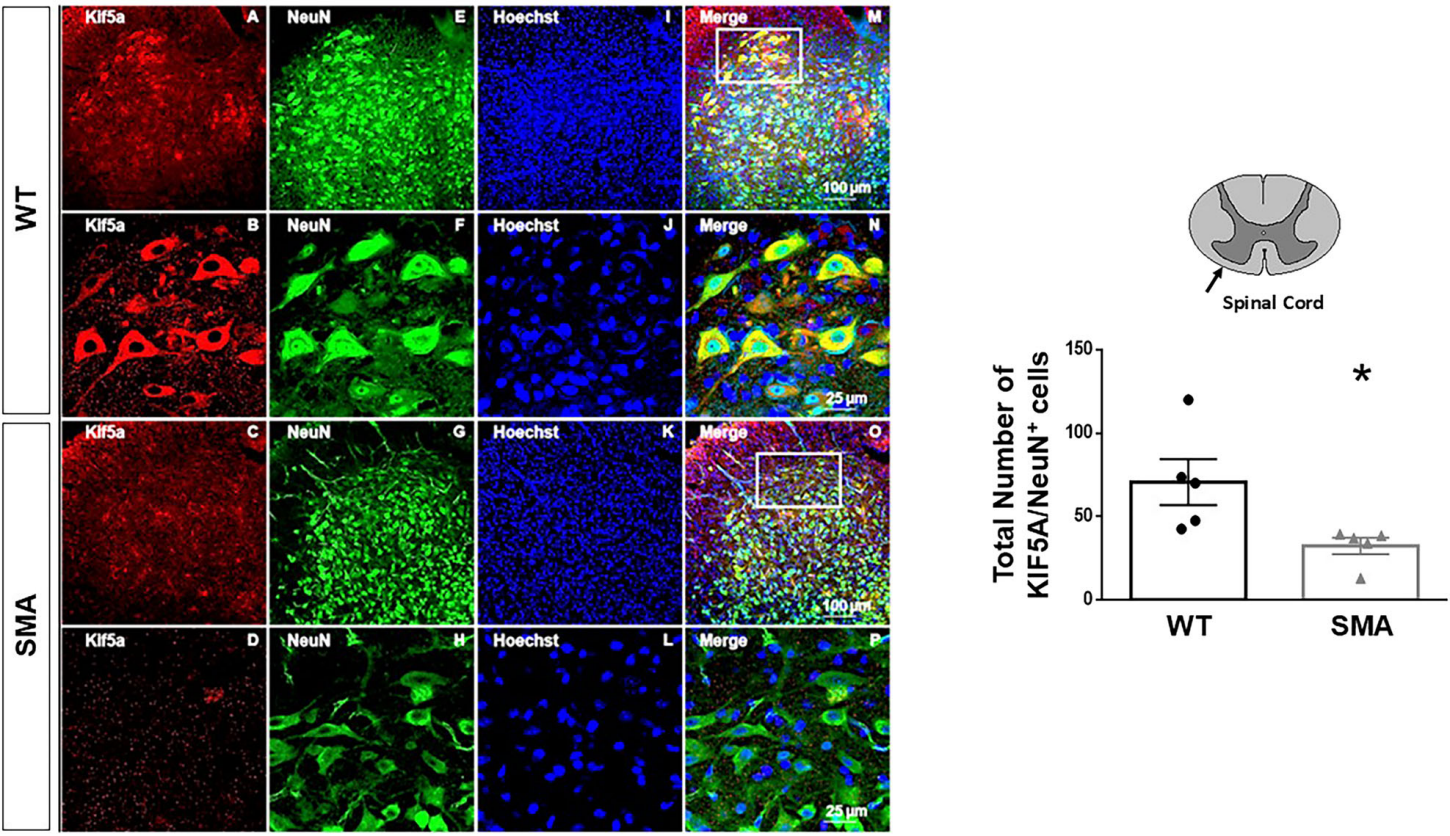
KIF5A expression in the lumbar spinal cord of late symptomatic SMA mice. Confocal images showing the double labelling of KIF5A (red) and NeuN (green) in lumbar spinal cord slices (40 μm thickness) of WT (**A**, **B**, **E**, **F**, **I**, **J**, **M**, **N**) and SMA (**C**, **D**, **G**, **H**, **K**, **L**, **O**, **P**) mice at P11. Nuclei were labelled with Hoechst (blu). The merge panels (**M**–**P**) showed the co-localization of KIF5A in neurons. Scale bar 100 μm for panels (**A**, **E**, **I**, **M**, **C**, **G**, **K**, **O**) and 20 μm for panels (**B**, **F**, **J**, **N**, **D**, **H**, **F**, **P**). The graph on the right represents the quantification of KIF5A fluorescence in neurons of WT (black dots) and SMA (grey triangles) mice,11 (P11) days after birth. Each column represents the mean ± SEM. Each point indicates a sample (*n* = 5). *p < 0.05 by Student’s t-test.

Afterwards, KIF5A localization was evaluated by confocal microscopy experiments in the spinal cord of WT and SMA mice in a late phase of the disease. In particular, the results obtained showed KIF5A downregulation in neuronal compartment. In fact, the total number of cells positive to the neuronal marker NeuN and to KIF5A, were strongly reduced in the spinal cord of SMA mice at P11 (Fig. 3), confirming WB results. Furthermore, double immunofluorescence experiments with specific marker for glial cells, such as GFAP or IBA1, did not show colocalization with KIF5A (data not shown), confirming the selective expression of KIF5A only in neurons, as previously reported [12].

### KIF5A gene is a novel target for miR-140-3p

**Fig. 4:**
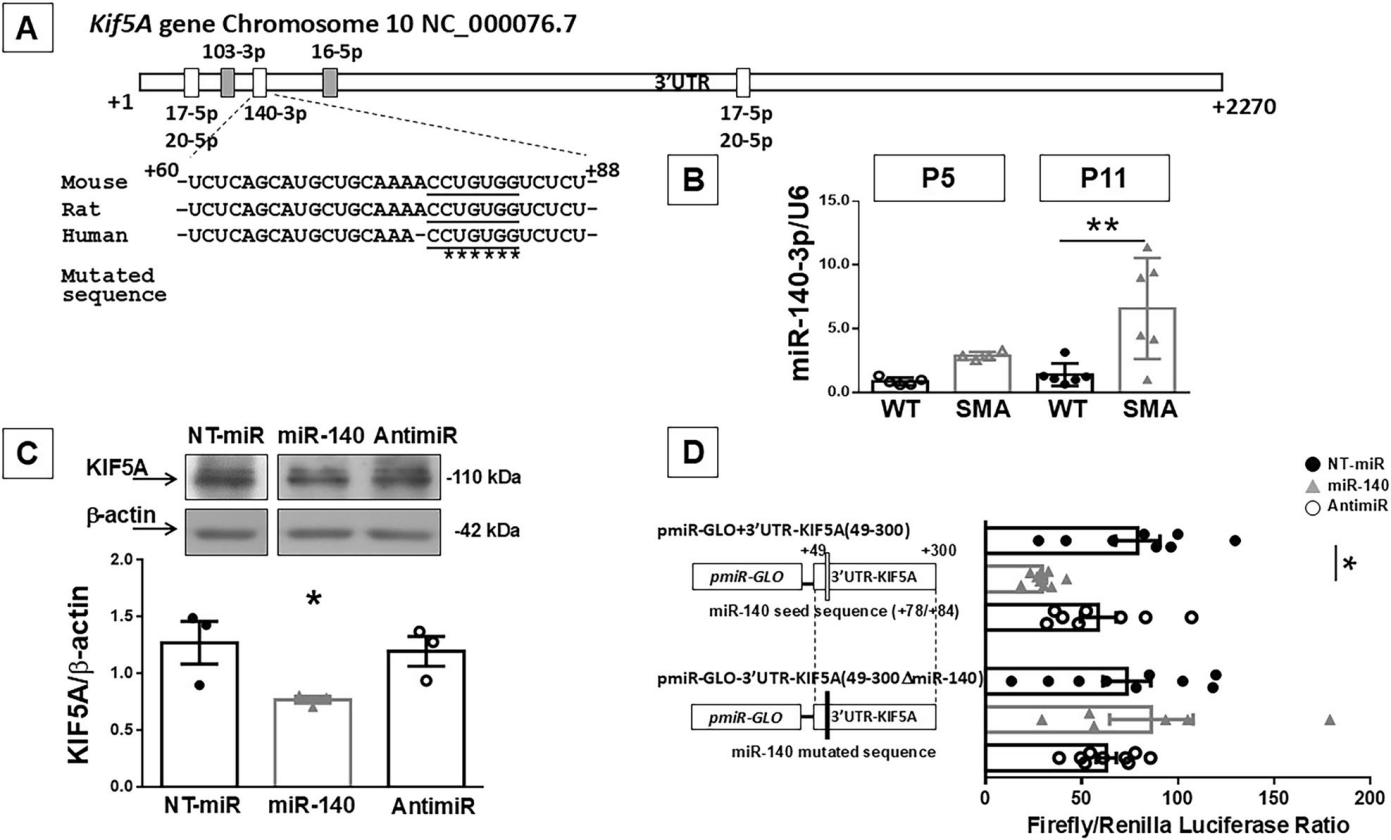
*Kif5A* gene is a new target of miR-140-3p. **A** Schematic representation of human 3’ UTR of *Kif5A* gene on chromosome 10. The rectangles (white and gray) represent the putative binding sites for several miRNA identified by TargetScan software (www.targetscan.org). Sequence alignment among mouse, rat and human miR-140-3p seed (underlined), showing a high percentage of conservation, was zoomed in. The nucleotides mutated in pmiR-GLO + 3’UTR-KIF5A(49-300ΔmiR-140) were indicated with a star. **B** Real time PCR for miR-140-3p in the spinal cord of WT (black dots) and SMA mice (gray triangles), 5 (P5, empty dots and triangles) and 11 (P11, full dots and triangles) days after birth. **p < 0.005 vs age-matched WT by one-way ANOVA analysis followed by Bonferroni test. **C** Western blot analysis of KIF5A protein in SH-SY5Y cells transfected with 50 nM of a non-targeting miRNA (NT-miR;black full dots), miR-140 (gray triangles) or a molecule able to block miR-140 (AntimiR;black empty dots). Panel D: Luciferase assay in SH-SY5Y cells co-transfect with pmiR-GLO + 3’UTR-KIF5A(49-300) or pmiR-GLO + 3’UTR-KIF5A(49-300ΔmiR-140) plus NT-miR, miR-140 or AntimiR. *p < 0.05 vs NT-miR by one-way ANOVA analysis followed by Bonferroni test. Each column represents the mean ± SEM. Each point indicates a sample.

In order to shed light on a neuroprotective strategy that could restore KIF5A levels in the spinal cord of SMA mice, we investigated the presence of potential miRNAs targeting the 3’ UTR region of *Kif5A* gene by TargetScan (www.targetscan.org). Interestingly, consensus sequences for miR-17-5p, miR-20-5p, miR-103-3p, miR-140-3p and miR-16-5p, highly conserved among vertebrates *H. sapiens, R. norvegicus* and *M. Musculus* were identified on *Kif5A* 3’ UTR (Fig. 4A). Therefore, we performed an initial screening of the expression level of these miRNAs in the spinal cord of P11 SMA mice, where we previously observed a significant downregulation of KIF5A. In particular, RT-PCR showed that miR-17-5p, -20-5p, -103-3p and miR-16-5p did not change their level of expression in SMA mice compared to age-matched controls (Fig. S1). By contrast, a significant 6.3-fold up-regulation of miR140-3p, that targets the position between +78/84 bp of *Kif5a* 3’ UTR was observed (Fig. 4B). Furthermore, an increase of miR140-3p levels of 3.3-fold were observed also at P5 (Fig. 4B). Thus, to investigate whether *Kif5A* gene can be a novel target for miR-140-3p, human neuroblastoma SH-SY5Y cells were transiently transfected with 50 nM of miR-140 mimic, or an inhibitor or a negative control with no homology to any mammalian gene. Forty-eight hours later, KIF5A protein levels were assessed by WB and the results indicated a significant 40% downregulation of this kinesin compared to the negative control (Fig. 4C). Furthermore, to confirm that *Kif5A* is a target of miR-140, a 251 bp region of *Kif5A* 3’ UTR that includes the miR-140 putative binding site at +78/84 bp, starting from the 49^th^ bp after the end of the open reading frame (ORF; indicated as +1), was cloned downstream of the firefly luciferase gene in the pmiR-GLO reporter vector (Fig. 4A). Then, SH-SY5Y cells were co-transfected with the pmiRGLO+3’UTR-*Kif5A* (49-300) construct and with NT-miR, miR-140, or AntimiR-140 molecules. Interestingly, when cells were co-transfected with miR-140, the luciferase assay showed a significant 65% reduction compared to NT-miR-transfected cells (Fig. 4D). On the other hand, co-transfecting miR-140 and a new mutated construct pmiR-GLO-3’UTR-Kif5A (49-300ΔmiR-140), in which the core sequence of miR-140 seed was mutated by site-directed mutagenesis (as shown in Fig. 4A), did not show any reduction in luciferase assay; demonstrating that miR-140-3p was able to: 1) bind a specific sequence on the 3’ UTR of *Kif5A* gene; 2) to modulate its expression.

### Intracerebroventricular administration of miR-140-3p ameliorates behavioural performance of SMA mice

In order to restore KIF5A levels in SMA mice, we intracerebroventricularly (icv) infused an AntimiRNA molecule, able to selectively blocks miR-140-3p function (AntimiR), to WT and SMA animals three times, at P1, P4, and P8 (50 pmol/1 μL per mouse), randomly dividing them into four experimental groups: WT + NT-AntimiR (Not Targeting AnitmiR),WT+AntimiR, SMA + NT-AntimiR, and SMA+AntimiR. To evaluate whether the AntimiR-140 could effectively reach spinal cord and brain stem regions and counteract miR-140 action, KIF5A protein levels were measured in these regions at P11 in WT animals icv-injected with the non targeting AntimiR or with the specific AntimiR molecule. Interestingly, a 50% increase in KIF5A protein was detected both in the spinal cord and in the brain stem of WT pups treated with the AntimiR (Fig. S2A, B) mice injected with the NT-AntimiR. Furthermore, immunofluorescence experiments were also performed to evaluate KIF5A modulation following AntimiR administration. Interestingly, the fluorescence intensity of cells positive to KIF5A was upregulated by 72% in the spinal cord of WT pups treated with the AntimiR at P11 compared to control ones (Fig. S3).

Thereafter, motor performance of WT + NT-AntimiR,WT+AntimiR, SMA + NT-AntimiR, andSMA+AntimiR mice were evaluated by exposing animals to a series of behavioural tests daily performed for 10 days, starting from the third day after birth (P3) to the 12^th^ day. The animals were then subjected to righting, negative geotaxis and tailing tests.

**Fig. 5:**
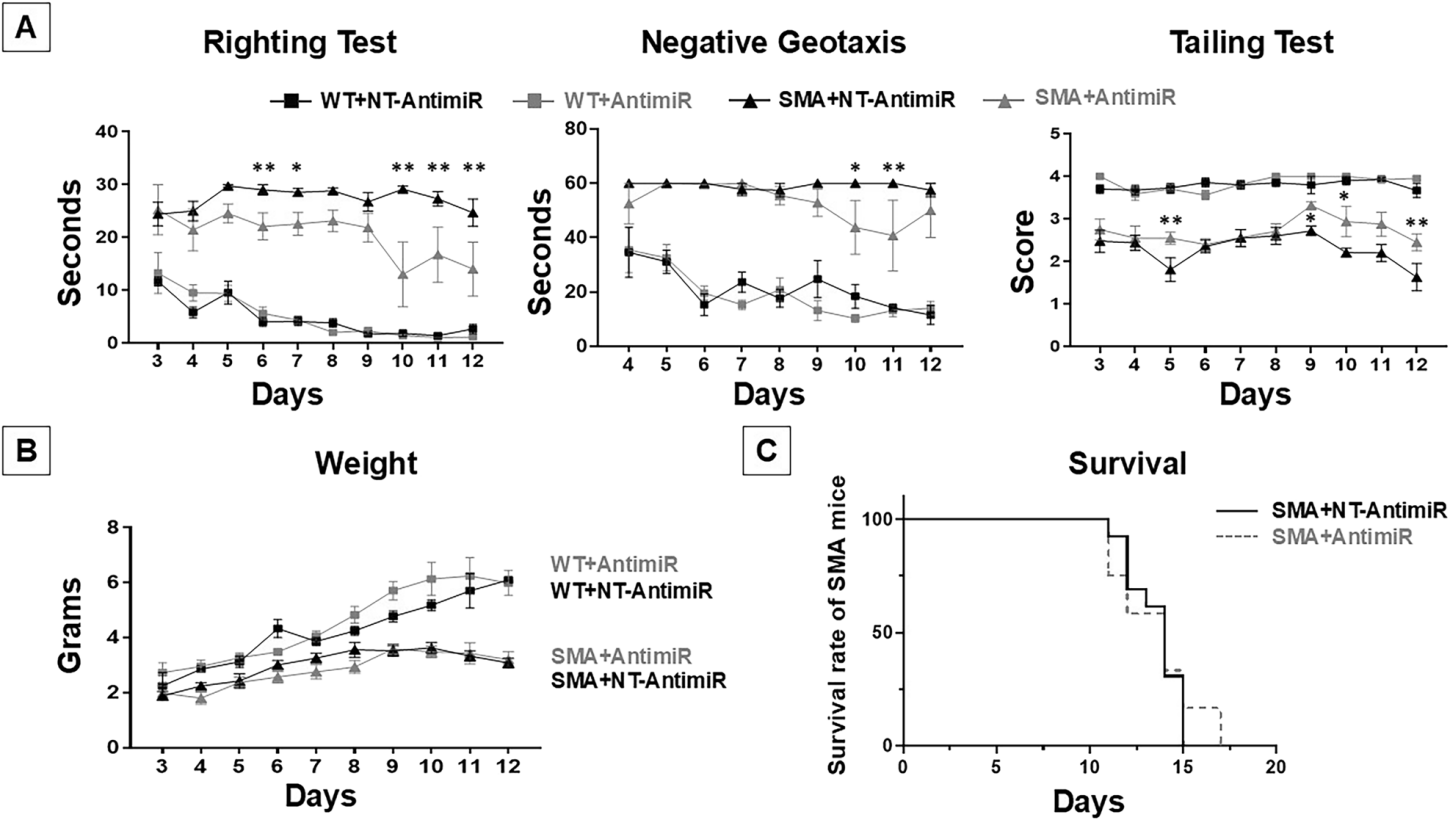
miR-140 administration ameliorated motor behavioral performances of SMA mice. **A** Righting, Negative Geotaxis and Tailing Suspension tests of WT + NT-AntimiR (a non-targeting AntimiR molecule; black squares), WT+AntimiR (a molecule able to block miR-140; gray squares), SMA + NT-AntimiR (black triangles), SMA+AntimiR (gray triangles) pups. *p < 0.05; **p < 0.005 SMA+AntimiR vs SMA + NT-AntimiR by 2-ways ANOVA analysis followed by Bonferroni test. **B** Growth curves of WT + NT-AntimiR,WT+AntimiR,SMA + NT-AntimiR, SMA+AntimiR pups. **C** Kaplan-Meier survival analysis expressed as percentage of SMA + NT-AntimiR (continuous line; *n* = 13) and SMA+AntimiR (dashed line, *n* = 12).

Motor coordination of WT and SMA pups treated with AntimiR or with NT-AntimiR was evaluated measuring the time the pups required, positioned upside down, to reposition themselves dorsal side up (30 sec was considered the cutoff time). SMA + NT-AntimiR pups were hardly able to complete the test, whereas SMA+AntimiR showed significant improvements starting at P6 (SMA+AntimiR, 22.1 ± 2.6 sec; versus SMA + NT-AntimiR, 29.0 ± 1.0 sec); at P7 (SMA+AntimiR, 22.6 ± 2.1 sec; versus SMA + NT-AntimiR, 28.6 ± 0.7 sec); at P10 (SMA+AntimiR, 13.0 ± 6.1 sec; versus SMA + NT-AntimiR, 29.1 ± 0.6 sec); at P11 (SMA+AntimiR, 16.8 ± 5.2 sec; versus SMA + NT-AntimiR, 27.3 ± 1.3 sec); and at P12 (SMA+AntimiR, 14.0 ± 5.1 sec; versus SMA + NT-AntimiR, 24.7 ± 2.6 sec) (Fig. 5A).

To evaluate both motor coordination and vestibular sensitivity, the mice were subjected to the negative geotaxis test. Briefly, animals were positioned on an inclined grid (approximately 35°) with the head facing down, and the time the mice spent to turn around with the head facing up was measured. At P4, the first day of test, WT + NT-AntimiR and WT+AntimiR pups were able to pass the test in 34.6 and 35.5 seconds, respectively (Fig. 5A). By contrast, SMA + NT-AntimiR usually failed this test with an average time of 60 s (cut-off time) both at P10 and P11. Interestingly, SMA+AntimiR pups were able to perform the test with an average time significantly shorter at P10 (43.8 ± 9.9 sec) and at P11 (40.8 ± 13 s) (Fig. 5A).

In order to further evaluate motor behavioural, mice were subjected to tailing-suspension test. This test assigns a score based on hindlimb posture when mice are suspended by the tail for 15 s. The highest value, 4, indicates a normal spreading of hind paws that decreases with self-clasping events that are usually observed in SMA animals. In fact, WT mice showed a score between 3.6 and 4 at all considered ages, with no significant differences between NT-AntimiR or AntimiR treated groups (Fig. 5A). On the other hand,SMA + NT-AntimiR pups showed a score ranging from 2.5 at P3 to 1.6 at P12, indicating hind limbs often close together. Interestingly, SMA+AntimiR mice showed a significant increase in hindlimb posture at P5 with a score of 2.6 ± 0.1, compared with age-matched SMA + NT-AntimiR pups (1.8 ± 0.3); at P9 (SMA+AntimiR, 3.3 ± 0.1; versus SMA + NT-AntimiR, 2.7 ± 0.1); at P10 (SMA+AntimiR, 2.9 ± 0.4; versus SMA + NT-AntimiR, 2.2 ± 0.1); and at P12 (SMA+AntimiR, 2.5 ± 0.2; versus SMA + NT-AntimiR, 1.6 ± 0.3)(Fig. 5A).

Finally we also evaluated SMA mice lifespan. However, no significant differences were observed in SMA pups lifespan treated with AntimiR or NT-AntimiR (Fig. 5C). Infact, the average lifespan of SMA + NT-AntimiR was of 13.5 ± 0.4 days, while that of SMA mice treated with AntimiR was of 13.6 ± 0.6 days (Fig. 5C).

## Discussion

In the present paper, taking advantage of a SMNΔ7 mouse model, we demonstrated for the first time that: 1) the mitochondrial transport kinesin KIF5A is strongly reduced in the spinal cord of SMA mice during disease progression; 2) a parallel upregulation of miR-140-3p levels takes place in the same CNS region; 3) icv injection in SMA mice of Antimir-140-3p ameliorates behavioural performance.

Axonal transport defects play a critical and, in some cases, a causative role in MNDs. In particular, KIF5A, a major component of the axonal transport machinery, has been associated with different MNDs including ALS and hereditary spastic paraplegia [30], but its role in SMA has not been fully characterized yet. Previous studies revealed a disruption of the anterograde as well as the retrograde axonal transport and reported reduced cargo transport in a SMNΔ7 mouse model [31]. Furthermore, mitochondrial distribution, transport and morphological defects are present in spinal MNs starting from the early stages of the disease [29].

Interestingly, analysis of different CNS regions affected by MNs degeneration such as brain stem, prefrontal cortex and spinal cord, at different phases of the disease, revealed a down-regulation of KIF5A protein levels in the spinal cord of both early and late symptomatic SMA mice. This suggests an alteration of axonal transport and possible of mitochondria, as they are almost exclusively transported via kinesin 1 [11]. Differently from the MIRO1 downregulation previously reported in ALS mouse models [32], the expression of the other members of the axonal transport complex, MIRO1, TRAK1 and TRAK2, was not altered in the spinal cord of SMA mice. In particular, MIRO1 is downregulated by a PINK1/Parkin-dependent degradation mechanism in SOD1 ALS mice [32, 33].

Interestingly, double immunofluorescence assay of lumbar spinal cord sections of SMA mice revealed an extensive neurodegeneration, a hallmark of SMA pathogenesis, and a general reduction of KIF5A staining in the remaining NeuN positive cells, specifically in the somatic motor nuclei. Overall, these results suggest that the disruption of the anterograde transport and possibly of other cargoes could be due to the loss of KIF5A complexes with MIRO1 and/or other adaptor proteins. It should be underlined that the other two isoforms of kinesin 1, KIF5B and C, could compensate for the reduction of KIF5A. Indeed, KIF5A and B can rescue the KIF5C null mice from a severe phenotype [13]. Moreover, there is a functional redundancy between the three KIF5s, not only due to the high homology in their sequence but also because they mostly bind cargoes through the highly conserved binding regions of kinesin light chains (KLC) [13]. However, mitochondria are transported with adaptor proteins TRAK1 and TRAK2, not KLC, and recent studies in the SC of SOD1-G93A mice showed a reduction only of KIF5A as the disease progresses [34], suggesting that the axonal transport defects observed in ALS are not rescued by an upregulation of KIF5B and C. Therefore, we assume that this could be the case also for SMA progression and tried to develop a miRNA strategy in order to restore KIF5A levels.

In particular, computational analysis of the 3’UTR region of *Kif5A* gene, highlighted the presence of putative consensus sequences for several miRNA highly conserved among vertebrates, miR-17-5p, miR-103-3p, miR-140-3p and miR-15-5p. An initial screening of the expression level of these miRNAs in the SC tissue of SMA mice, where the most dramatic downregulation of KIF5A was observed, revealed a significant increase only of miR-140-3p. This miRNA-RNA interaction, characterized by a stringent 7mer-m8 site type, was confirmed by a luciferase reporter assay carried out in SH-SY5Y cells. Indeed, co-transfection of miRNA-140-3p with the luciferase construct containing the wild type miRNA site, resulted in a decrease of luciferase activity. Although the mouse *Kif5a* 3’ UTR length is over 2500 bp, we cloned only a small region containing the predicted target sequence. In this way, we investigated the specific interaction of the miR-140-3p mimic with its own site, limiting non-specific effects that could occur with the whole 3’UTR. Moreover, endogenous miR-140-3p levels in SH-SY5Y cells were also assessed and found to be at extremely low levels or undetectable compared with the miRNA-mimic transfected cells. Therefore, effects of the endogenous miRNA on the luciferase expression can be considered irrelevant. Therefore, miR-140-3p is capable to downregulate KIF5A expression in vitro thus suggesting that manipulation of miR-140-3p levels could be an effective strategy in preventing KIF5A downregulation. Indeed, the icv infusion of AntimiR-140-3p in vivo, induced an up-regulation of KIF5A expression in the spinal cord of SMA mice. As a consequence, AntimiR-140-3p ameliorated behavioural performances of SMA mice. Interestingly, SMA pups usually fail both at righting and negative geotaxis tests, while SMA treated with the AntimiR molecule were able to perform these tests in a significant shorter time than SMA + NT-AntimiR animals. Furthermore, tailing test showed that AntimiR-treated animals could spread wider their hind limbs compared to untreated ones. On the other hand, AntimiR administration did not significantly improve weight and lifespan of SMA pups. Probably, addressing only axonal transport, and specifically mitochondrial one, is not enough to counteract SMN deficiency. Indeed, mitochondrial dysfunctions and oxidative stress have long been reported in patients with SMA [27, 28] together with a significant reduction of mitochondrial transport, numbers and size in axons of SMA spinal MNs [29] as early neuropathological features [25]. Therefore, we suggested that axonal transport deficit, mitochondrial dysfunctions and oxidative stress, might be targeted at the same time to obtain a significant amelioration to alleviate the burden of the disease.

Moreover, we hypothesised that SMN could be a novel cargo of KIF5A for two main reasons. First, SMN is actively transported along the axons at rates consistent with fast axonal transport [35]. Secondly, SMN overexpression in SOD1 and TDP-43 mouse models, known to be affected by axonal transport impairment and KIF5A reduction, resulted in a perinuclear accumulation of SMN, limiting its beneficial effects on the phenotype [36]. This could be an important aspect to investigate as if confirmed, this will be important not only to understand the axonal role of SMN and the contribution of axonal RNA trafficking and local translation defects in SMA but also in other MNDs. In fact, we know that low levels of SMN predispose to MNs cell death in SMA and ALS, whereas healthy MNs are characterized by normal levels of SMN [37, 38] There are currently several studies suggesting a significant overlap between SMA and ALS and a broader role of SMN in MNDs [37, 39]. With SMN-elevating strategies currently available [40] it would be particularly interesting to investigate whether restoring not only the levels but also the transport and axonal localisation of SMN via KIF5A could be beneficial in SMA and in other MNDs.

For all the above-mentioned reasons we believe that KIF5A has a central role in MNDs as therapeutic and diagnostic toll, and that developing a strategy able to prevent KIF5A downregulation could be beneficial not only in SMA but also in other neurodegenerative diseases.

## Materials and Method

### Animal model

The original breeding *Smn*^*+/−*^*; hSMN2*^*+/+*^*; hSMNΔ7*^*+/+*^ mice, heterozygous and healthy carrier for *Smn* gene mutation, were purchased from Jackson Laboratory (stock number 005025; Jackson Laboratories) and were bred to obtain *Smn*^*+/+*^*; hSMN2*^*+/+*^*; hSMNΔ7*^*+/+*^ (WT) animals and *Smn*^*−/−*^*; hSMN2*^*+/+*^*; hSMNΔ7*^*+/+*^ (SMA) animals. The colony was maintained by interbreeding carrier mice, and the offspring were genotyped by PCR assays on tail DNA according to the protocols provided by Jackson Laboratory. This mouse model, SMNΔ7, is one of the most common used to study SMA at the preclinical level. Mice at birth are smaller than normal littermates and have a lifespan of about 13 days and impairment of motor behaviour clearly detectable 4–5 days after birth (P4–P5) [41]. Therefore, mice were sacrificed at P5 and P11, considering P0 as the day of birth. Overall, 34 WT mice and 38 SMA mice, housed under diurnal lighting conditions (12 h darkness/light) were used. Experiments were performed according to the international guidelines for animal research and approved by the Animal Care Committee of “Federico II” University of Naples, Italy and Ministry of Health, Italy. All efforts were made to minimize animal suffering and to reduce the number of animals used.

### RT-PCR analysis

Mice were deeply anesthetized with 3% isoflurane vaporized in O_2_/N_2_O 50:50 and sacrificed. Brain and spinal cord were rapidly removed and immediately frozen on dry ice and stored at −80 °C until use. Total RNA was extracted following supplier’s instructions (Life Technologies, Monza, Italy) and cDNA was synthesized using 0.5 or 2 μg of total RNA to obtain miRNA specific cDNA or total cDNA, respectively, with the High-Capacity Transcription Kit following supplier’s instruction (Life Technologies). Quantitative real-time PCR was performed with TaqMan assays in a 7500 real time PCR system (Life Technologies). Changes in mRNA and miRNA levels were determined as the difference in threshold cycle (2^-ΔΔCt) between the target genes (*Kif5A* ID: Mm00515265_m1; *Rhot1* ID: Mm01304158_m1; *Trak1* ID: Mm00613053_m1; *Trak2* ID: Mm00549615_m1) or miRNA-140-3p (ID:002234) and the reference gene: beta-glucuronidase (*Gusb*) for mouse tissues (ID:Mm00446953_m1), glyceraldehyde-3-phosphate dehydrogenase (*Gapdh*) for cell tissues (ID:Mm99999915_g1) and U6 snRNA (ID:001973) for miRNA-140-3p, as previously reported [42].

### Western blot

Total extracts were obtained as previously described [43]. To detect the proteins of interest, specific antibodies were used: anti-KIF5A (rabbit polyclonal antibody, 1:1000; Life Technologies); anti-MIRO1 (rabbit polyclonal antibody, 1:1000; Life Technologies); anti-TRAK1 (rabbit polyclonal antibody, 1:1000; Life Technologies); anti-TRAK2 (rabbit polyclonal antibody, 1:1000; Life Technologies), and mouse monoclonals antiβ-actin (1:1000; Sigma-Aldrich, Milan, Italy). Immunoreaction was revealed using anti-mouse and anti-rabbit immunoglobulin G conjugated to peroxidase 1:10000 (GE Healthcare, Norwalk, CT) by the ECL reagent (GE Healthcare). The optical density of the bands was determined by Chemi Doc Imaging System (Biorad, Milan, Italy) and normalized to the optical density of β-actin, used as internal controls.

### Immunofluorescence analysis and cell counting analysis

Immunostaining and confocal immunofluorescence procedures were performed as previously described [44]. Briefly, animals were anesthetized and transcardially perfused with saline solution containing 0.01 ml heparin, followed by 4% paraformaldehyde in 0.1 mol/l PBS saline solution. Spinal cord was rapidly removed on ice and postfixed overnight at +4 °C and cryoprotected in 30% sucrose in 0.1 M phosphate buffer (PB) with sodium azide 0.02% for 24 h at 4 °C. Next, Spinal cord was sectioned frozen on a sliding cryostat at 40 μm thickness, in rostrum-caudal direction. Afterwards, free floating serial sections were incubated with PB Triton X 0.3% and blocking solution (0.5% milk, 10% FBS, 1% BSA) for 1 h and 30 min. The sections were incubated overnight at +4 °C with the following primary antibodies: anti-NeuN (mouse monoclonal antibody; 1:1000; Millipore, Milan, Italy), anti-KIF5A (1:500; Life Technologies). The sections were then incubated with the corresponding florescent-labeled secondary antibodies, Alexa 488/Alexa 594 conjugated anti-mouse/anti-rabbit IgGs (Molecular Probes, Invitrogen, Milan, Italy). Nuclei were counterstained with Hoechst (Sigma-Aldrich). Images were observed using a Zeiss LSM700 META/laser scanning confocal microscope (Zeiss, Oberkochen, Germany). Single images were taken with an optical thickness of 0.7 m and a resolution of 1024 × 1024. In double-labeled sections, the pattern of immune reactivity for both antigens, was identical to that seen in single stained material. The number of KIF5A^+^ and NeuN^+^ positive cells was determined by manual counting at 20× magnification in the spinal cord of WT and SMA mice.

KIF5A fluorescence intensity quantification on tissue sections at spinal cord level, was assessed in terms of pixel intensity value, using the NIH image software as previously described [45]. Briefly, digital images were taken with 40 × objective, and identical laser power settings and exposure times were applied to all the photographs from each experimental set. Images were first thresholded to identify the positive signal; subsequently, the pixels expressing KIF5A were identified. Finally, the number of pixels positive for KIF5A was measured per microscope field. Images from the same areas of each region were compared. For manual counting of NeuN and KIF5A, five mice per group were included in the analysis. For the fluorescence analysis four mice per group and 3 sections for each genotype were used. Results were expressed in arbitrary units [46].

### Cloning of the human Kif5A 3’untraslated region (3’UTR)

Polymerase chain reaction amplification was performed using PrimeSTAR^®^ GLX DNA polymerase (Takara, Kusatsu, JP) on mouse cDNA, priming from *Kif5A* stop codon at +3601 bp to +3860 bp (GenBank accession number NM_001039000.4), inserting a XhoI and a NotI restriction site within the forward and the reverse primers, respectively, designed using the OligoPerfect tool (Life Technologies). The PCR product was purified using StrataPrep DNA Gel Extraction Kits (Agilent, Milan, Italy) and cloned into multiple cloning sites of pmirGLO Dual-Luciferase miRNA Target Expression Vector (Promega, Milan, Italy) downstream of the firefly luciferase gene (*luc2*). The primer sequences flanked by XhoI and NotI sites used for the amplification were: *Kif5A*-3’UTR fwd:5’-ccgctcgagaggcctcttctctcagcatg-3’and *Kif5A*-3’UTRrev: 5’ttgcggccgcaaagaagaaatattccctcctccc-3’. The fidelity of the constructs was confirmed by DNA sequencing. Site-directed mutations were carried out in the miR-140-3p binding seed sequence using the Q5 site-directed mutagenesis kit (New England Biolabs, Ipswich, MA, USA) according to the manufacturer. Desired mutations were confirmed by sequencing. The primer used for site-directed mutagenesis were: *Kif5A*-3’UTR mut fwd: 5’-gctgcaaaacgatatctctctgacactaactccctccc-3’ and *Kif5A*-3’UTR mut rev:5’- atgctgagagaagaggcc-3’that inserted an EcoRV restriction site, useful for colony screening.

### Cell cultures, transient transfection and reporter assay

Human neuroblastoma SH-SY5Y cells (ATCC, Manassas, VA, USA) were maintained in Dulbecco modified eagle medium supplemented with 10% FBS, 1% L-Glutammine, 1% Penicillin-Streptomycin and were plated 24 h before transfection.

For WB assay, SH-SY5Y cells were transfected with 50 nM of hsa-miR-140-3p mimic (miR-140; MC12503, Qiagen) or the negative control (NT-miR; 1027280, Qiagen) or specific miRNA inhibitor (AntimiR; MH12503, Qiagen) with Lipofectamine 2000 (Invitrogen) according to the manufacturer protocol. Forty-eight hours after transfection cells were harvested.

For dual luciferase assay, SH-SY5Y cells were co-transfected with 100 ng of each DNA plasmids (the pmirGLO-3’UTR, pmirGLO-mutated 3’UTR), in combination with 30 nM of miR-140-3p mimic or the NT-miR or AntimiR. Forty-eight hours after transfection cells were harvested following the supplier’s instructions (Promega) and the light emitted was measured with a manual luminometer (Glomax 20/20, Promega). The effect of 3′-UTR activity on the reporter gene was calculated as firefly-to-renilla ratio and normalized for the luciferase of the empty vector.

### Stereotaxic surgery and administration of AntimiR-140-3p

Intracerebroventricular (icv) administration of 1 μl of LNA modified AntimiRNA-140-3p (AntimiR; miRCURY LNA miRNA Inhibitor, Qiagen, Milan, Italy) or of a non-targeting AntimiRNA (NT-AntimiR; miRCURY LNA miRNA inhibitor control, Qiagen) at the concentration of 50 μM at P1, P4, P8 was performed as previously described [47]. Briefly, neonatal mice were anesthetized by hypothermia (total duration 2’) and their heads were immobilized on a custom neonatal stereotaxic apparatus at 4 °C during surgery. AntimiR or NT-AntimiR molecules were injected at stereotaxic coordinates of 0 mm from bregma, 0.8 mm lateral to sagittal sinus, and 1.5 mm deep with a Hamilton microsyringe. The pups were then placed on a heat pad, quickly revitalized and subsequently returned to their mother.

### Behavioral assessment

In order to analyze the progression of motor symptoms in WT and SMA mice treated with AntimiR or with NT-AntimiR, the animals were divided in four experimental groups: WT + NT-AntimiR (*n* = 13); WT+AntimiR (*n* = 13), SMA + NT-AntimiR (*n* = 16) and SMA+AntimiR (*n* = 14), and daily checked body weight and performed behavioral tests, starting at P3 as previously reported [47, 48].

Righting reflex: pups were placed on their backs on a flat surface. The time employed to right themselves was recorded (cut off 30 s). Tail suspension (self-clasping test): pups were suspended by the tail for 15 s and their hindlimb posture was scored. The score was: 4, hindlimbs spread open; 3, hindlimbs not completely spread; 2, hindlimbs often close together; 1, hindlimbs always close together; 0, hindlimbs always close together with additional postural abnormalities (clasping). Negative geotaxis from P4: the animals were placed on an inclined grid (at approximately 35° inclination) with the mouse head facing down. We measured the time needed for mice to orient themselves (cut off 60 s). This test is useful to evaluate both motor coordination and vestibular sensitivity.

### Statistical analysis

Data were evaluated as means ± SEM. Statistically significant differences among means were determined by one-way ANOVA followed by Bonferroni post-hoc test for western blotting, cell counting, real time PCR and luciferase analysis. Two-way ANOVA followed by Bonferroni post-hoc was used for motor performances test and body weight analysis. The Kaplan-Meier plot was used to evaluate survival. Student’s t-test was used for two groups comparison. Statistical significance was accepted at the 95% confidence level (*p* < 0.05). Statistical analyses were performed by using GraphPad Prism 5.0 (La Jolla, CA, USA). All experiments were carried out in a blind manner.

## Supplementary information

Supplementary File

Original WB data

## Data Availability

All data supporting the findings of this study are available within the paper and its Supplementary Information.
