# Supplementary material for: MiR-140-3p regulates axonal motor protein KIF5A and contributes to axonal transport degeneration in SMA
Source: Cell Death Discov. 2025 Oct 7;11:446. doi: 10.1038/s41420-025-02663-x (PMC12504656; doi:10.1038/s41420-025-02663-x)
Supplement: Supplementary file 1 — Supplementary File [file 41420_2025_2663_MOESM1_ESM.pdf]

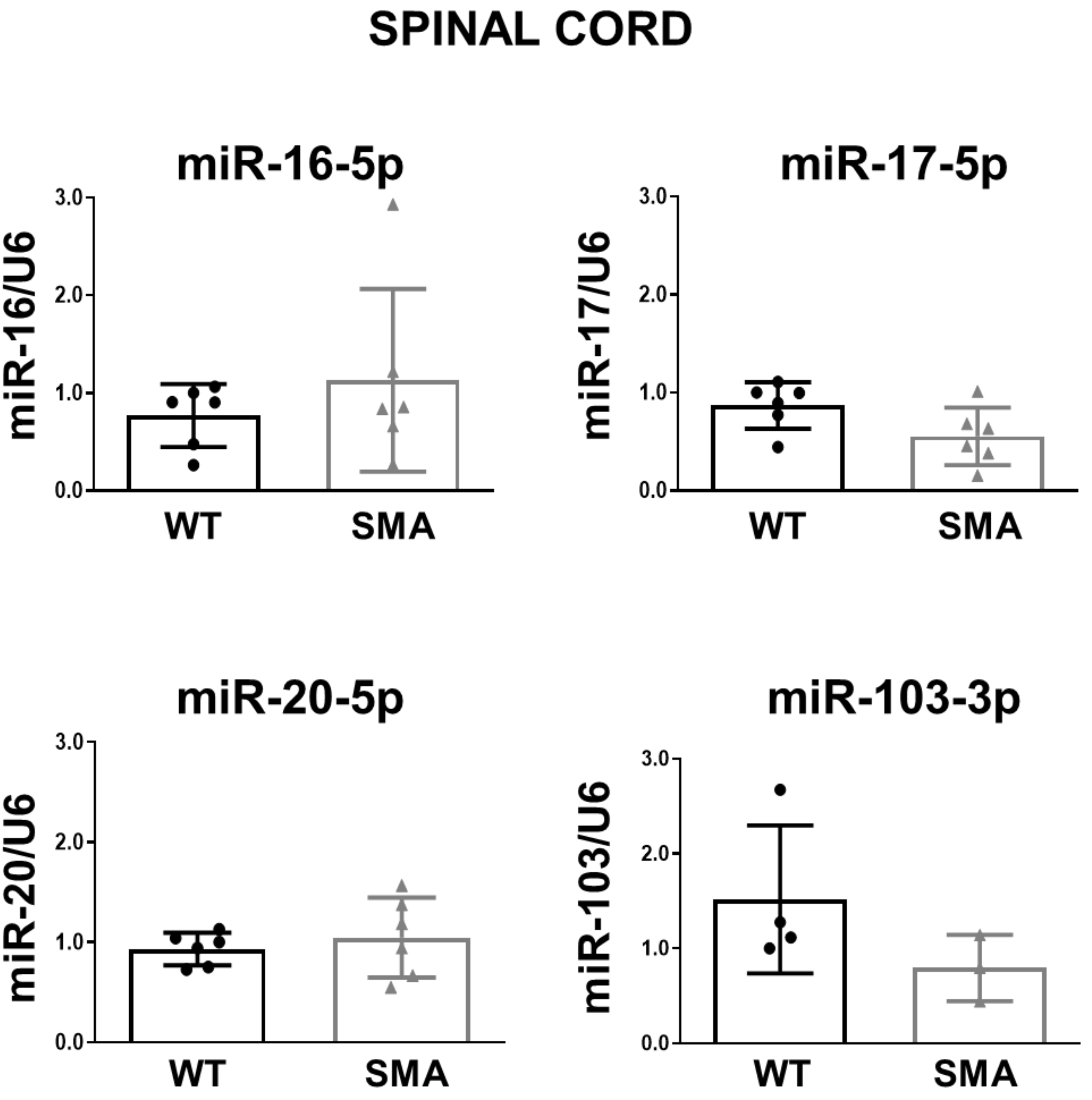

Figure S1. miR-16, miR-17, miR-20and miR-103expression levels in the spinal cord of late symptomatic SMA mice. Real time PCR for miR-16-5p, -17-5p, 20-5p and -103-3p in the spinal cord of WT (black dots) and SMA (grey triangles) mice,11 days after birth. Each column represents the mean ± SEM. Each point indicates a sample.

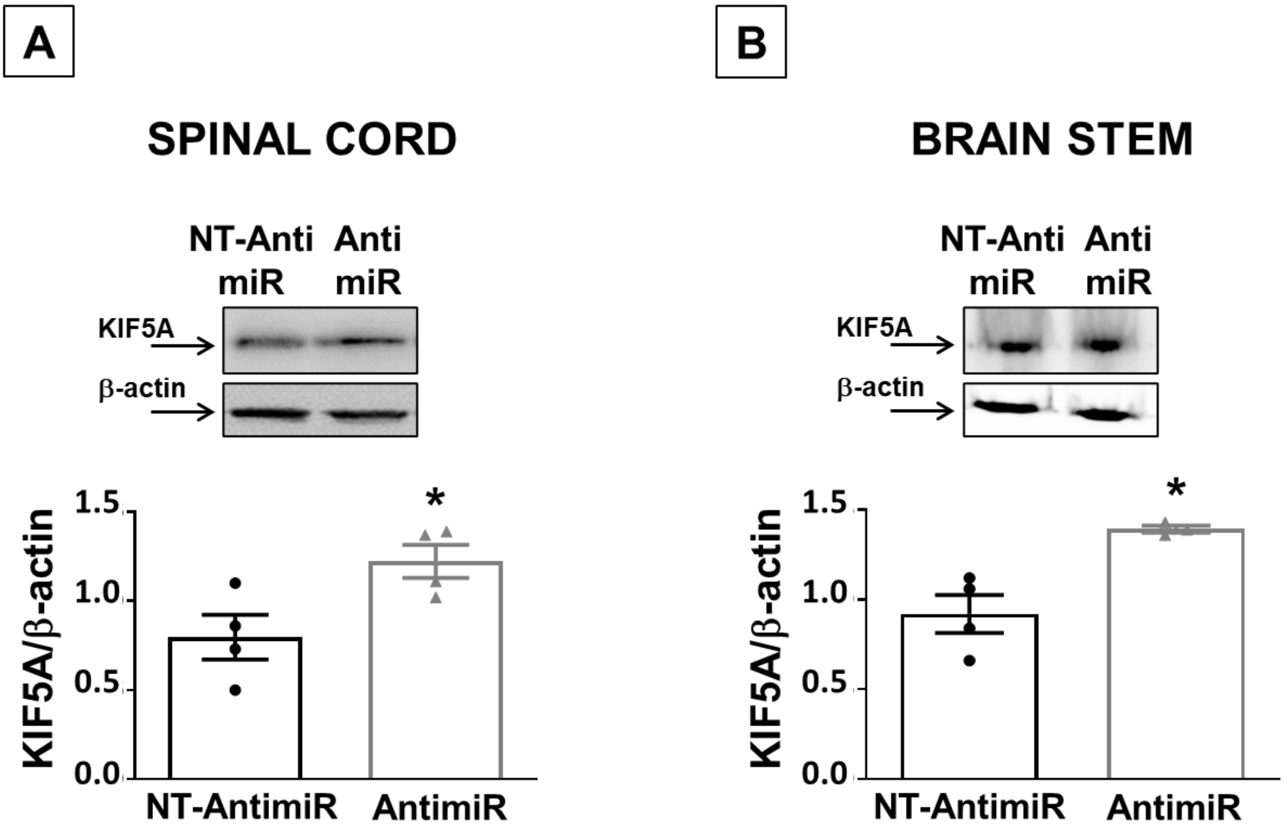

Figure S2. KIF5A protein expression in the spinal cord and in the brain stem of SMA mice after icv injection of AntimiRmolecule. Western blot analysis for KIF5A protein in the spinal cord (A) and in the brain stem (B) of WT+NT-AntimiR (a non-targeting AntimiR molecule; black dots), and WT+AntimiR (a molecule able to block miR-140; gray triangles) icv-injected mice, 11 days after birth. Each column represents the mean ± SEM. Each point indicates a sample. *$p<0.05$ WT+AntimiR vs WT+NT-AntimiR by Student's t-test.

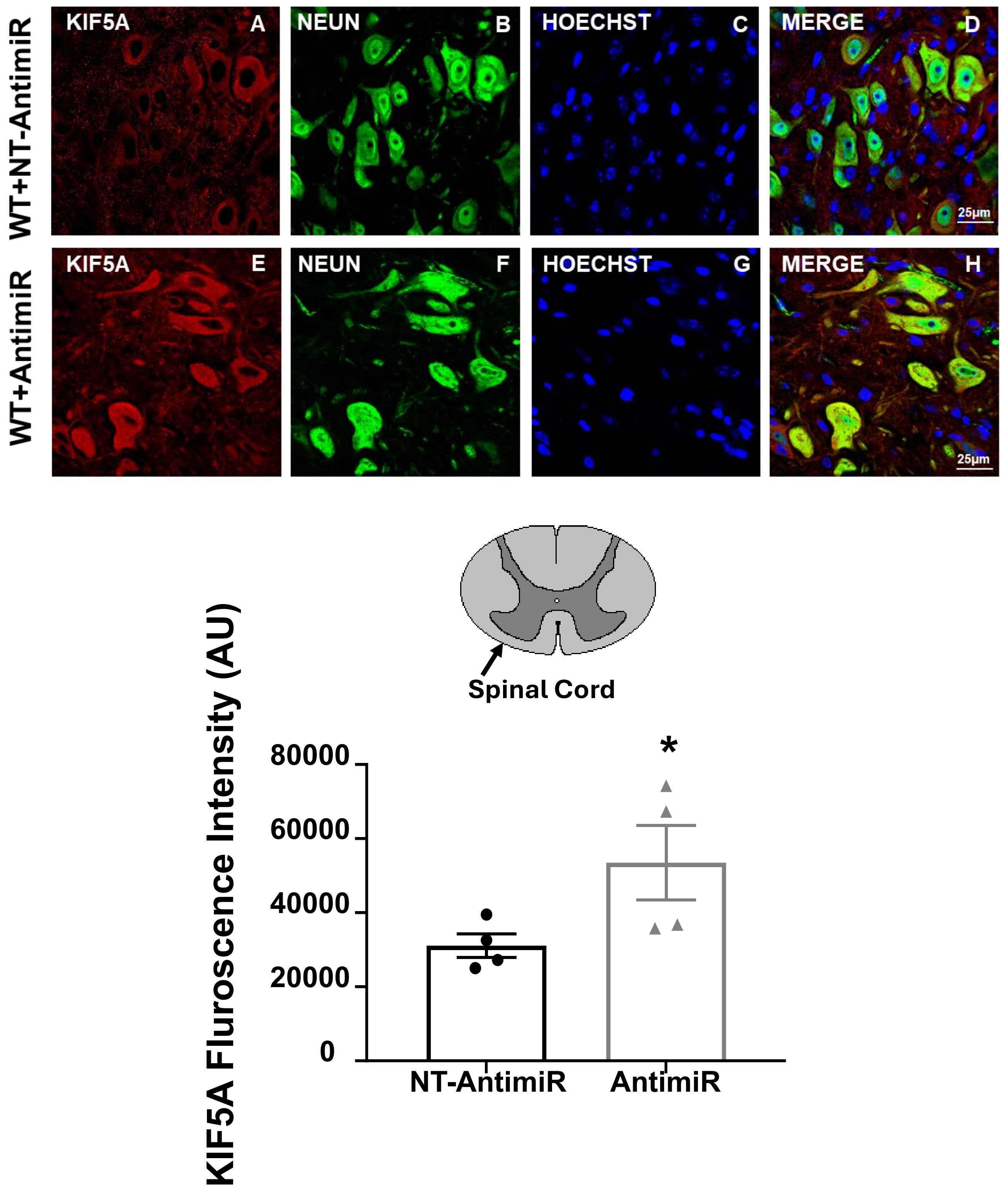
WT+NT-AntimiR
WT+AntimiR
KIF5A
A
NEUN
B
HOECHST
C
MERGE
D
25μm
KIF5A
E
NEUN
F
HOECHST
G
MERGE
H
25μm
Spinal Cord
KIF5A Fluroscence Intensity (AU)
80000
60000
40000
20000
0
*
NT-AntimiR
AntimiR

Figure S3. KIF5A immunoflorescence analysis in the spinal cord of WT mice after icv injection of AntimiR molecule. Confocal images showing the double labelling of KIF5A (red) and NeuN (green) in lumbar spinal cord slices (40 mm thickness) of WT mice treated with NT-AntimiR (A, B, C, D) or AntimiR (E, F, G, H) molecules at P11. Nuclei were labelled with Hoechst (blu). The merge panels (D, H) showed the co-localization of KIF5A in neurons. Scale bar 25mm for all panels. The graph on the right represents the quantification of KIF5A fluorescence intensity per photographic field (mm^2), arbitrary units (AU) for WT+NT-AntimiR (black dots) and WT+AntimiR (grey triangles) mice, at P11. Each column represents the mean ± SEM. Each point indicates a sample (n=4). *$p<0.05$ by Student's t-test.
