# Supplementary material for: MiR-140-3p regulates axonal motor protein KIF5A and contributes to axonal transport degeneration in SMA
Source: Cell Death Discov. 2025 Oct 7;11:446. doi: 10.1038/s41420-025-02663-x (PMC12504656; doi:10.1038/s41420-025-02663-x)
Supplement: Supplementary file 2 — Original WB data [file 41420_2025_2663_MOESM2_ESM.pdf]

Figure 1 A

SPINAL CORD P5 WB
del 20/5/2020

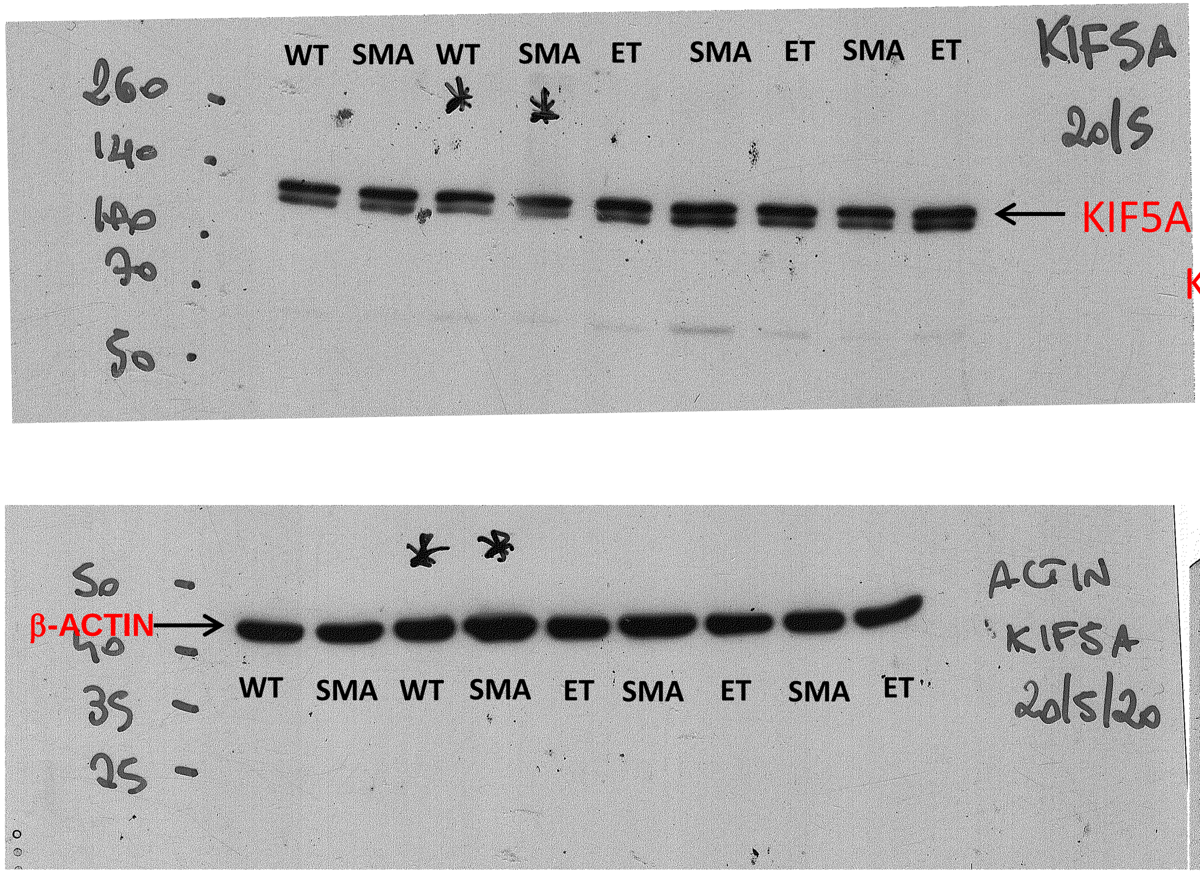

M- 46/3 - 47/2 - 46/5 - 47/3 - 47/4 - 49/0 - 47/0 - 49/1 - 46/4

Figure 1 A

SPINAL CORD P10
WB del 25/2/2021

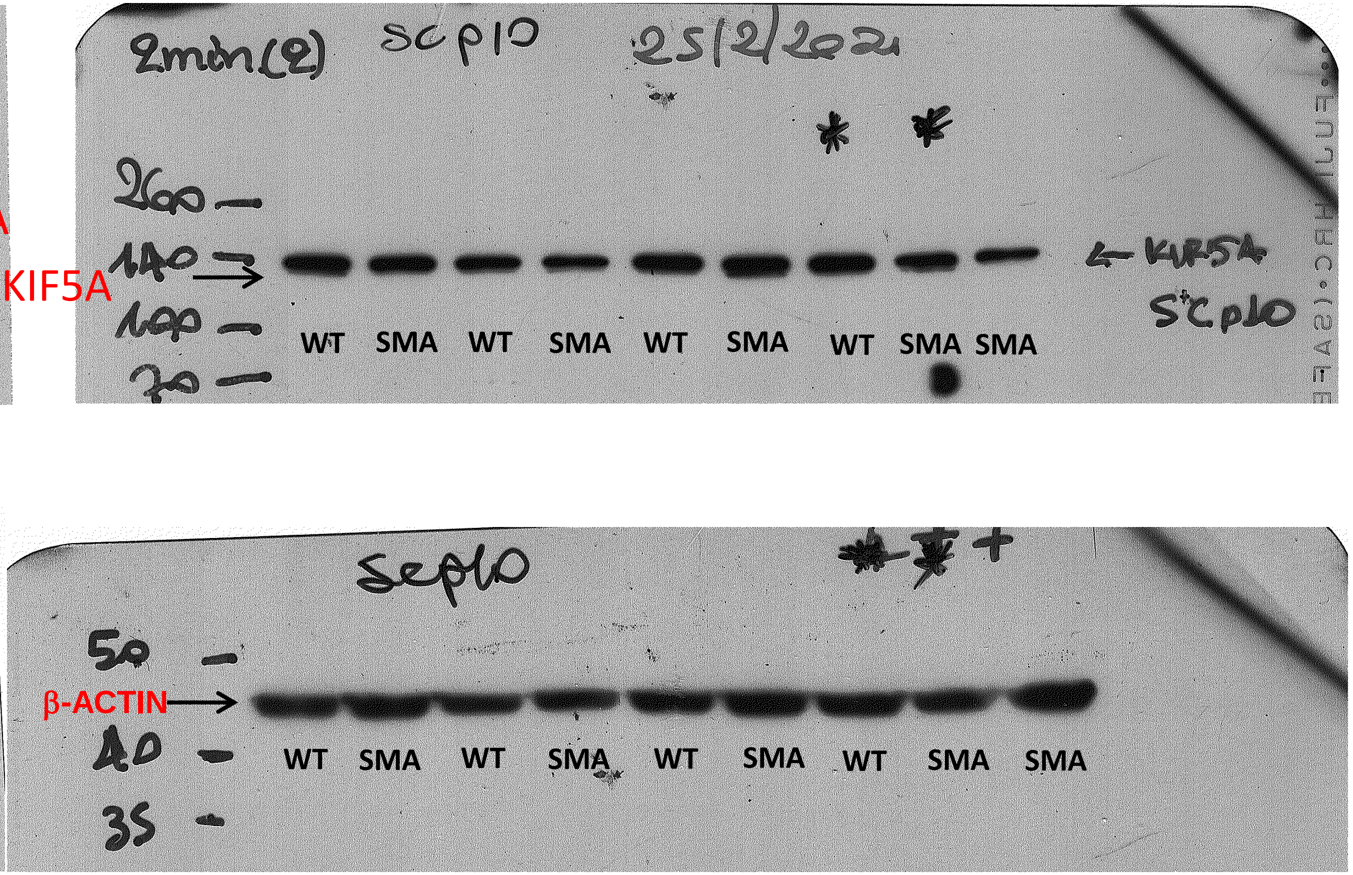

M- 68/5-68/1-97/4-68/2-106/1-68/3-106/5-100/0-106/0

Figure 1 B

PREFRONTAL CORTEX P5 WB del 25/11/2020

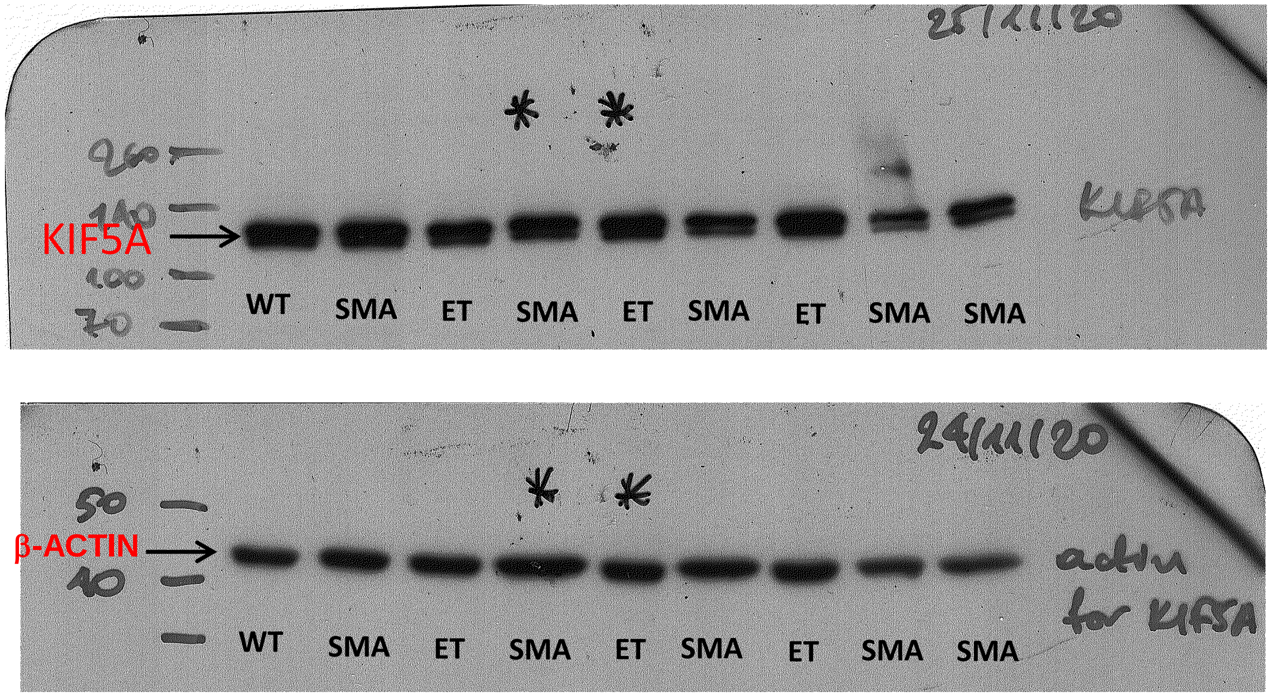

M- B12/5-A1/0-B2/1-B2/0-B2/5-A5/0-A5/5-B12/0-B12/1

Figure 1 B

PREFRONTAL CORTEX P10 WB del 20/01/2021

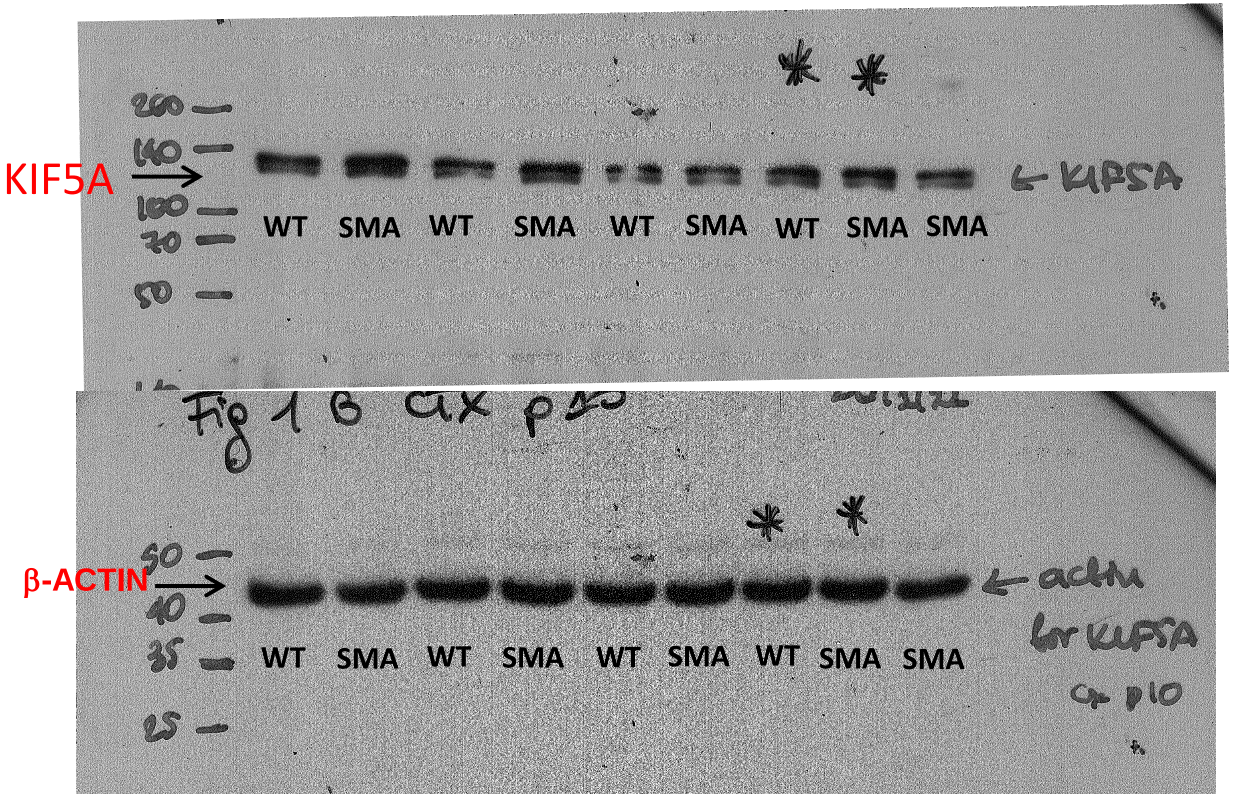

M- 79/0-85/0-80/1-92/0-92/6-79/5-92/7-80/0-84/0

Figure 1 C

BRAINSTEM P5 WB
del 29/01/2020

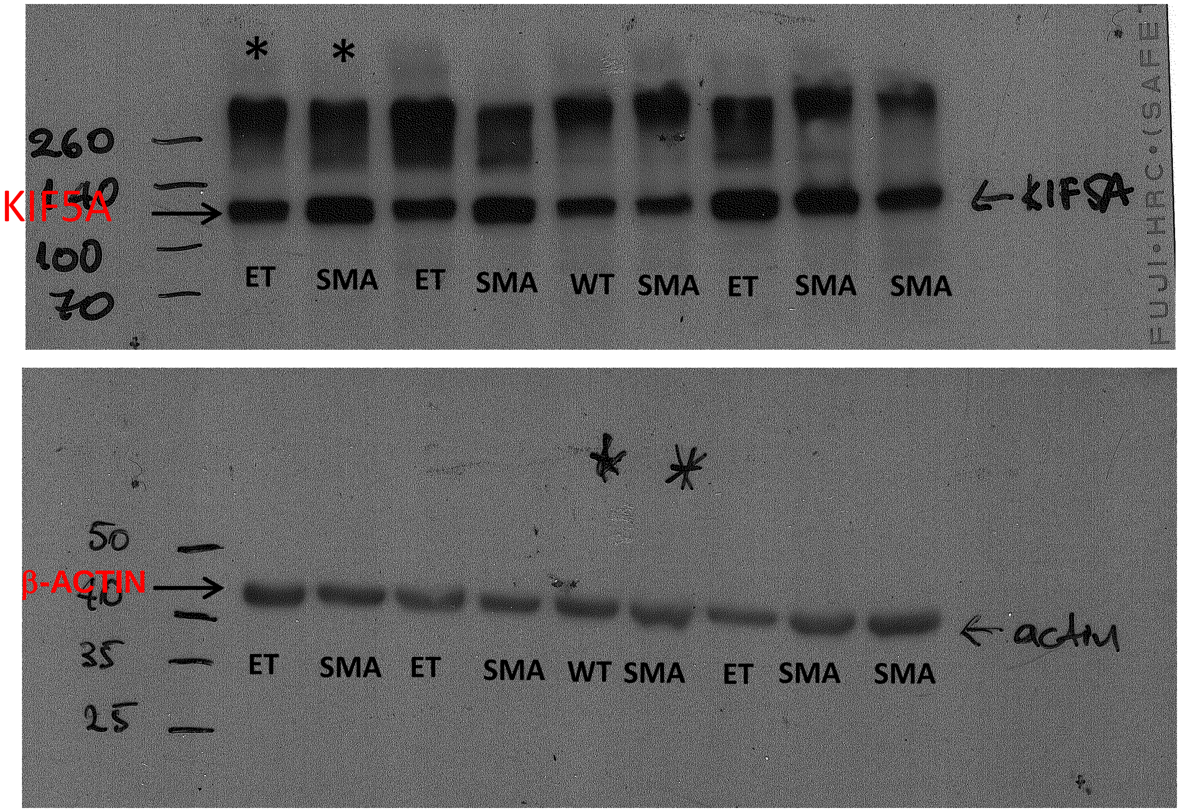

M-B2/1-B2/0-A5/1-A5/0-B12/5-B12/0-A5/5-B12/1-A1/0

Figure 1 C

BRAINSTEM P10 WB
del 14/05/2020

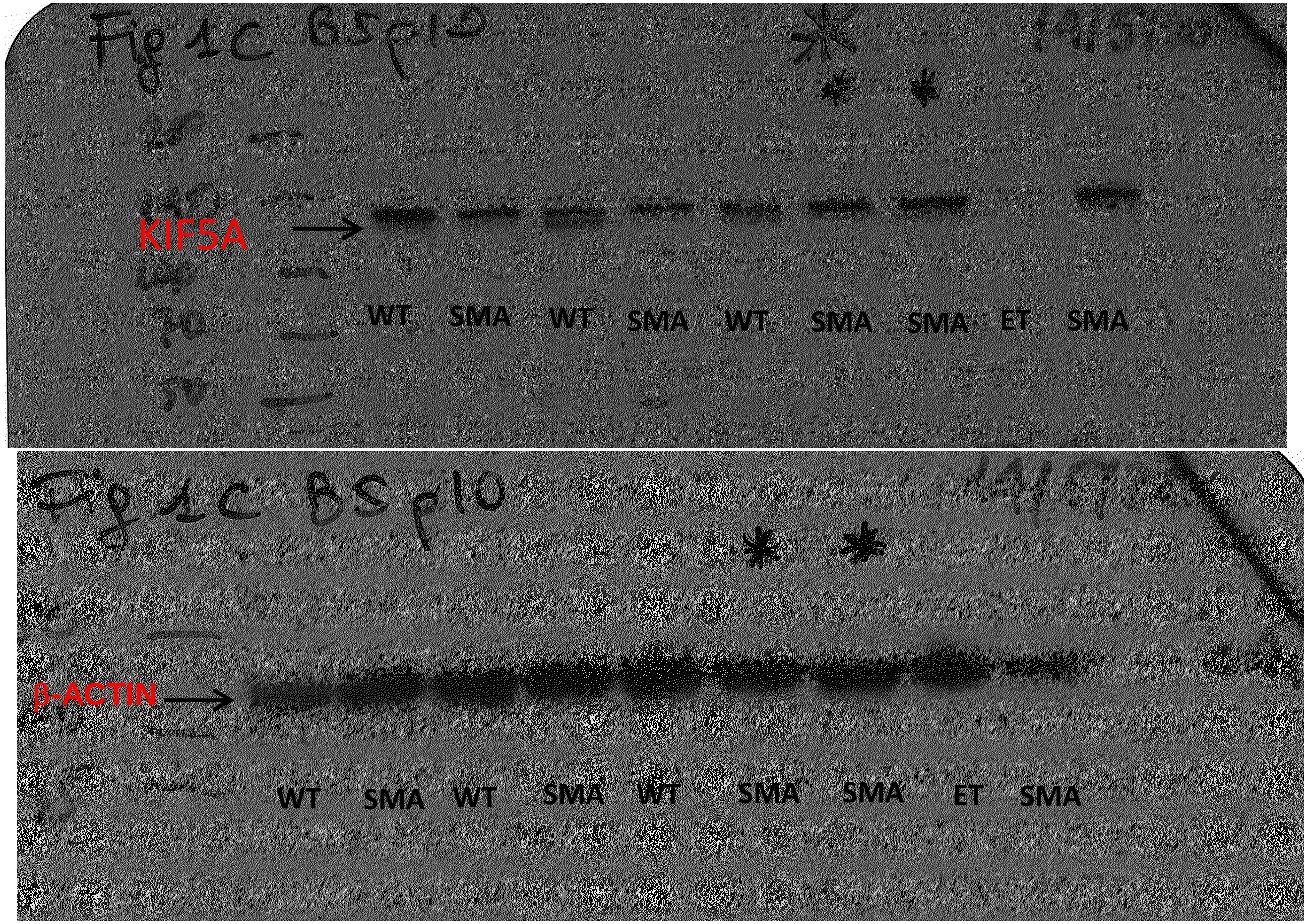

M-41/2-37/2-46/9-37/3-46/8-41/0-41/1-37/4-42/0

Figure 2 A

SPINAL CORD P5 WB
del 20/05/2020

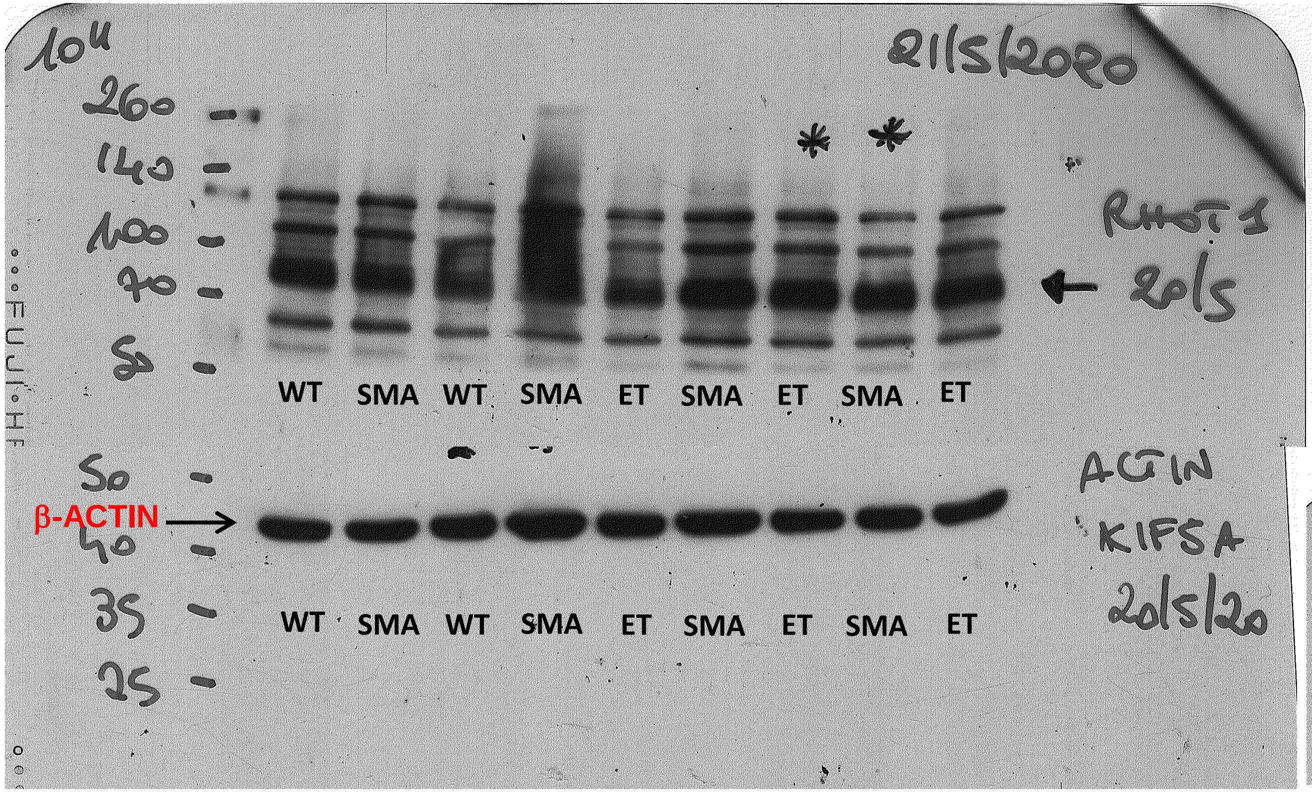

Figure 2 A

SPINAL CORD P10
WB del 26/2/2021

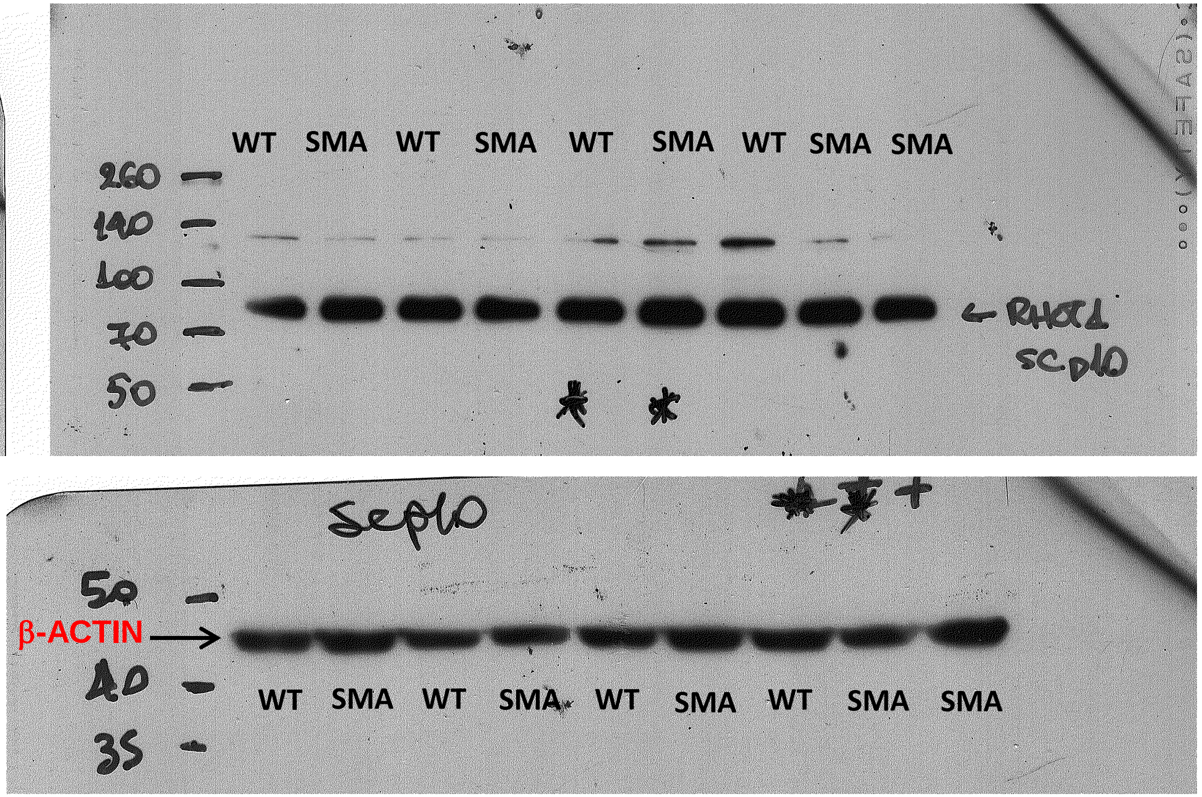

M- 46/3 - 47/2 - 46/5 - 47/3 - 47/4 - 49/0 - 47/0 - 49/1 - 46/4

M- 68/5-68/1-97/4-68/2-106/1-68/3-106/5-100/0-106/0

Gel strippato di KIF stessa actina fig 1a SC p5

Figure 2 B

SPINAL CORD P5 WB
del 08/06/23

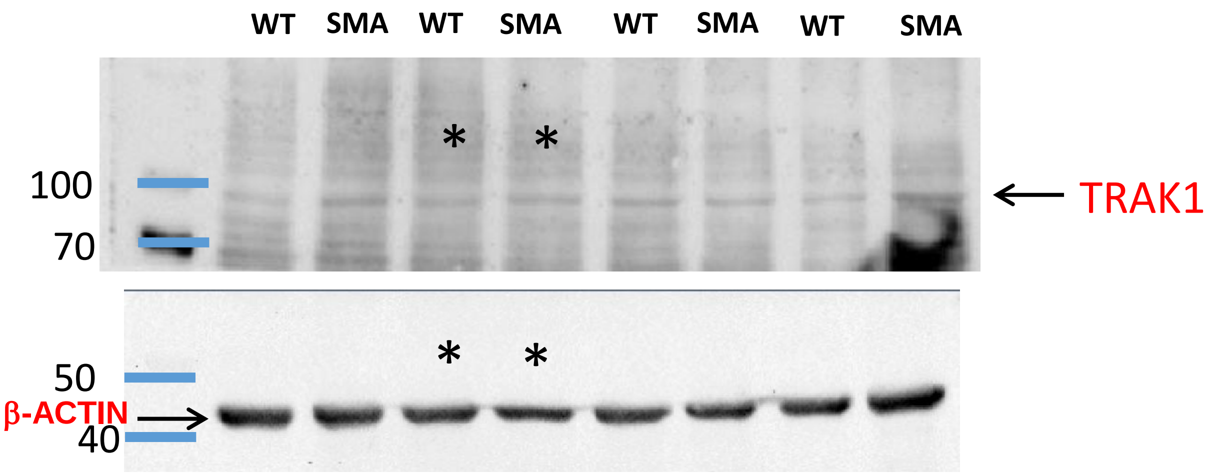

M- 240/5-241/6-240/10-241/20-242/1-243/0-242/6-244/0

Figure 2 B

SPINAL CORD P10
WB del 21/06/23

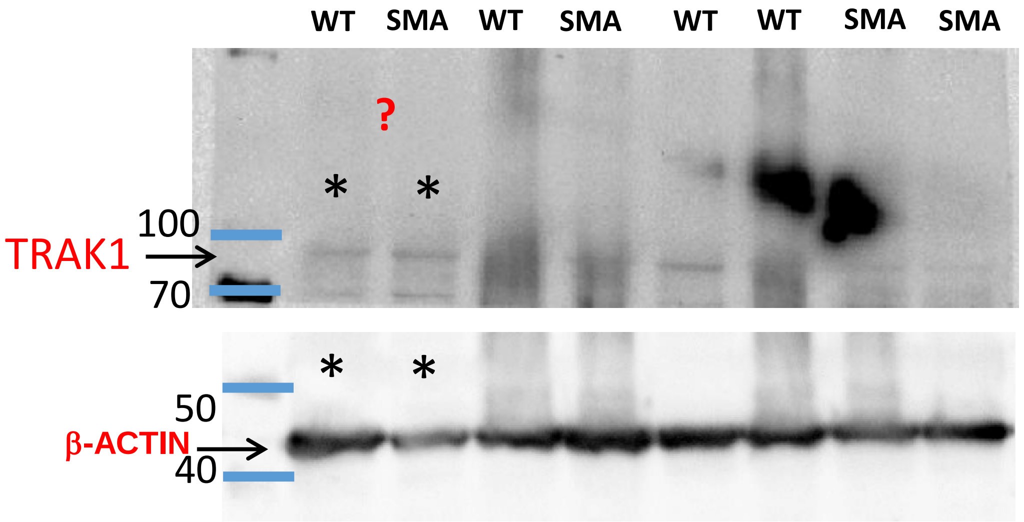

M- 219/10- 219/1 – 219/50-220/0-220/1-220/5-217/0-219/0

Figure 2 C

SPINAL CORD P5 WB
del 08/06/23

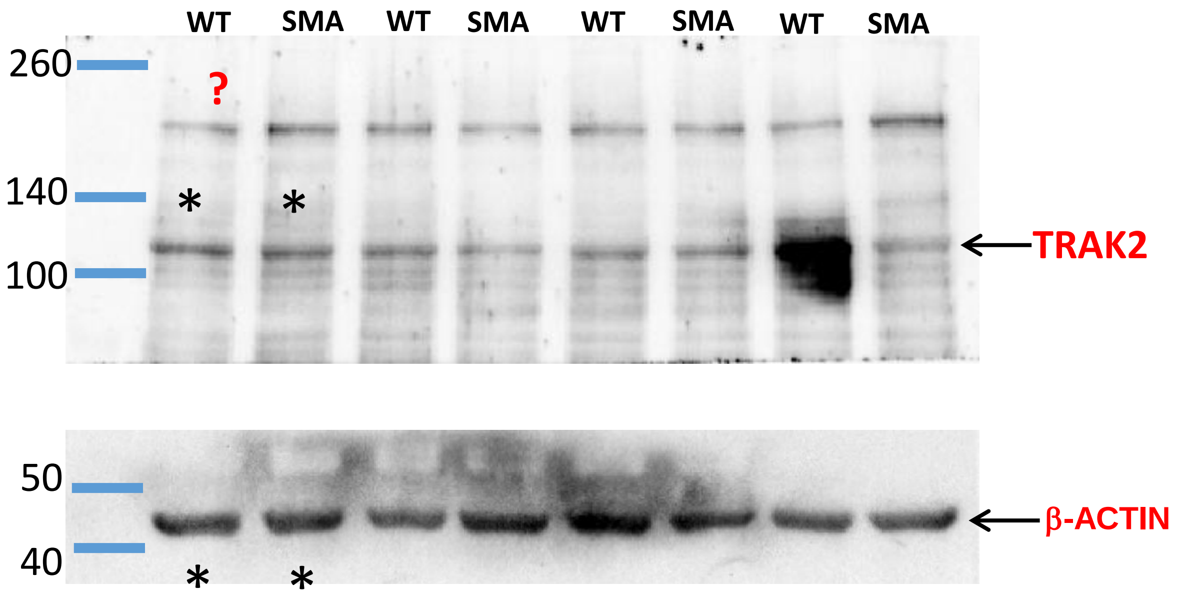

M- 240/5-241/6-240/10-241/20-242/1-243/0-242/6-244/0

Figure 2 C

SPINAL CORD P10
WB del 21/06/23

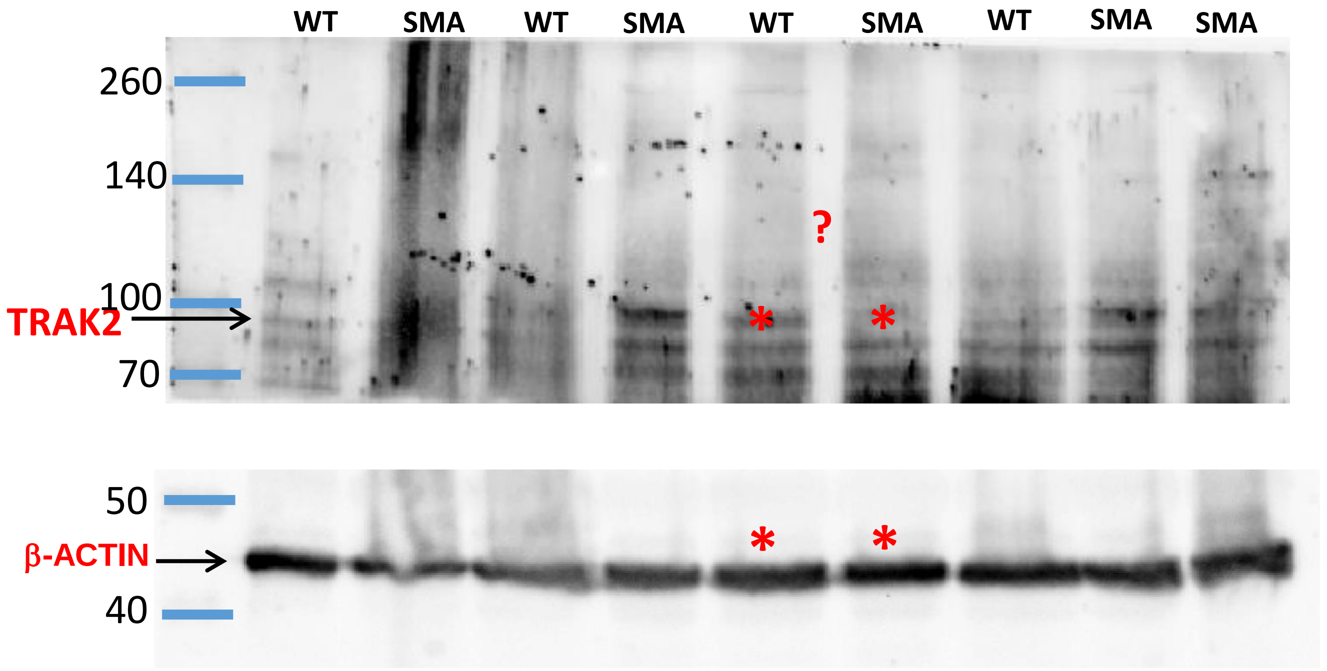

M- 219/10- 219/1 – 219/50-220/0-220/1-218/0-220/5-217/0-219/0

Figure 4 C

WB del 9/2/2022

(SH-SY5Y transfected 31/01/2022)

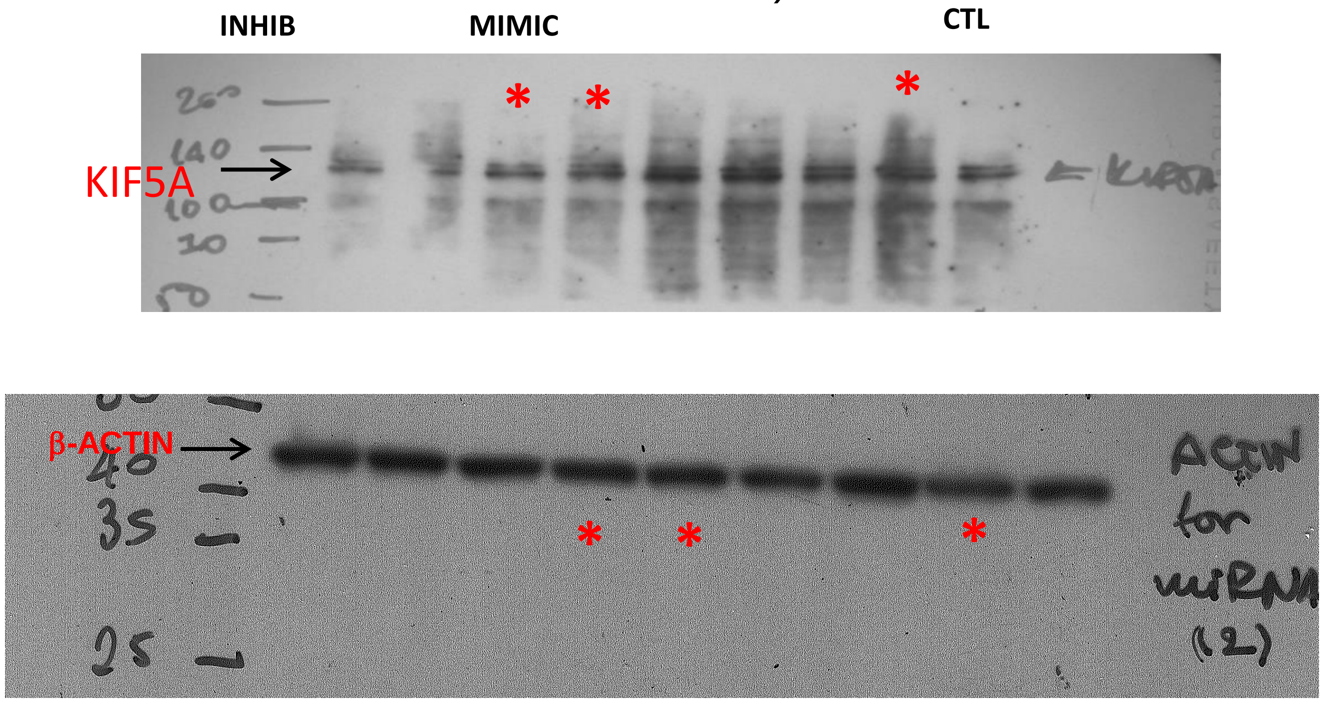

Figure S 2

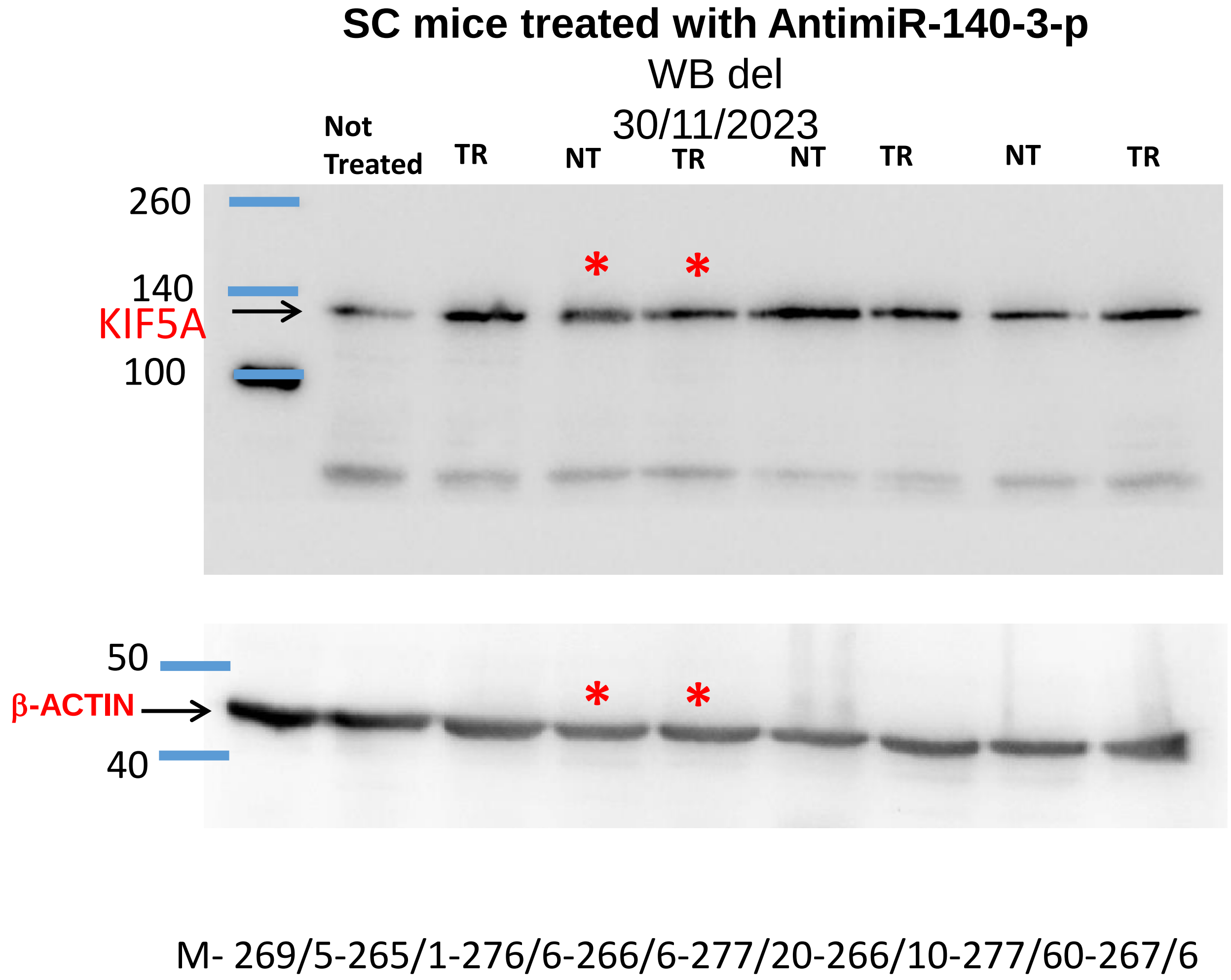
SC mice treated with AntimiR-140-3-p
WB del
30/11/2023
Not Treated
TR
NT
TR
NT
TR
NT
TR
260
140
KIF5A
100
*
*
50
β-ACTIN
40
*
*
M- 269/5-265/1-276/6-266/6-277/20-266/10-277/60-267/6

BS p12 antimiR administration

Loading order: M- TG – TG+antimiR – TG - TG+antiMiR - TG – TG+antimiR

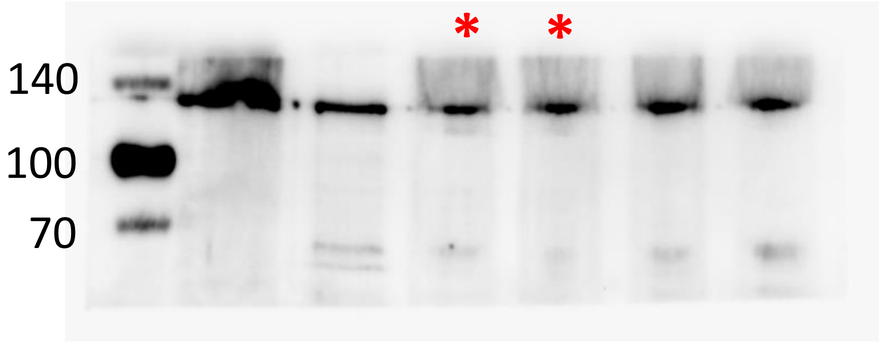

KIF5A 120 KDa

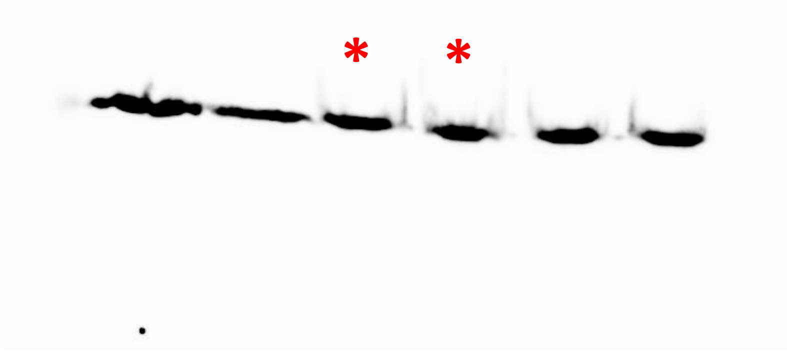

ACTIN 42 KDa
